# (Mis)matched direct and moderating relationships among pro-environmental attitudes, environmental efficacy, and pro-environmental behaviors across and within 11 countries

**DOI:** 10.1371/journal.pone.0304945

**Published:** 2024-06-18

**Authors:** Lindsay B. Miller, Ronald E. Rice

**Affiliations:** Department of Communication, University of California Santa Barbara, Santa Barbara, Santa Barbara, California, United States of America; Aalto University, FINLAND

## Abstract

Pro-environmental behaviors are influenced by individuals’ pro-environmental attitudes and environmental efficacy, among many other factors. However, attitude-behavior models are inconsistent on whether and how attitudes, efficacy, and behaviors should match in specificity or generality, and on the moderation effect of efficacy. This study first tests a simple model including direct and moderating relationships between pro-environmental attitudes, environmental efficacy, and pro-environmental behaviors. Then it examines relationships among subscales matched or mismatched in their respective specific or general domain of environmental attitudes (concern, values), environmental efficacy (self, collective), and pro-environmental behaviors (private, public). Secondary data come from an overall sample of 11,000 respondents across 11 countries, with n = 1,000 from each country. Pro-environmental attitudes and efficacy have direct relationships with pro-environmental behavior, but efficacy has little moderation effect. Different combinations of (mis)matched measures produce slightly different results, with the most variance explained, counter to hypotheses, by two mismatched models. Results are generally consistent across countries.

## 1 Introduction

As issues of environmental degradation become increasingly pressing and publicized [1, 2], it is imperative that scholars have a clear understanding of the forces that can lead individuals to engage in pro-environmental behaviors. Gifford [3] underscores that “understanding behavior at the psychological level of analysis… is essential, given that the cumulative impact of individuals’ decisions and behaviors is the key factor driving climate change” (p. 554). Two such antecedents that have been widely studied are pro-environmental attitudes and efficacy. Although ample research has explored the relationships among pro-environmental attitudes, efficacy, and behavior [e.g., 4], the field lacks a coherent understanding of the many subdimensions of these constructs and the relationships among them. Furthermore, there is little research that explores how these relationships may persist or vary across countries—an understanding of which is vital to combat such global phenomena.

Copious research has examined the association of pro-environmental attitudes with pro-environmental behaviors. Across behavior types, ages, and nationalities, people’s pro-environmental attitudes are positively related to their intended and enacted pro-environmental behaviors [e.g., 5–14]. However, other studies report weak or non-significant relationships [5, 15, 16]. These inconsistent findings suggest that the environmental attitude-pro-environmental behaviors relationship is complicated by at least two conceptual issues.

The first issue is the extent to which the pro-environmental attitude-behavior relationship is conditioned by additional influences. One such influence is *efficacy*. When individuals perceive that they cannot perform a given action, or that their performance of that action is not likely to succeed, intentions and behavior weaken [17–19]. Requiring further clarification is whether efficacy operates as a direct effect on (is associated with) pro-environmental behavior and/or as a moderator of the pro-environmental attitude-behavior relationship.

The second issue is the extent to which measures of pro-environmental attitudes, environmental efficacy, and pro-environmental behaviors should be *specific*, *general*, *or combined*. Previous research suggests that large discrepancies occur when the specificity of attitude measurement does not match the specificity of the respective behavior [20–22]. For example, Kaiser [23] notes inconsistent results when using ecological behavior measures that do not explicitly consider the nature of the behaviors, or their correspondence to measured attitudes. Thus “an often-recommended means to increase consistency is *measurement correspondence*, that is, measuring both attitude and behavior on the same level of specificity” (p. 398). The theory of planned behavior (TPB) and related research argues that stronger relationships occur when measures of both attitudes and behaviors are specific [17, see also 24, concerning green purchases]. Similarly, Heeren et al. [15] analyzed the relationships between attitudes, knowledge, norms, and perceived behavioral control on each of 10 sustainability behaviors, finding much more variance was explained when the behaviors were analyzed separately (more specific) than when combined into one scale (more general). Indeed, some specific pro-environmental attitude variables seem to be associated with or affect only specific pro-environmental behaviors [25, p. 416].

However, Kaiser [23] and Kaiser et al. [26] argued that both pro-environmental attitudes and pro-environmental behaviors should be measured generally, partially because so many specific influences and challenges vary across individuals and contexts. Many researchers follow this approach by using measures of global environmental attitudes in their research [e.g., 23, 27] (see Sections 2.1, 6.2).

Further, this unidimensional measurement of pro-environmental attitudes and pro-environmental behaviors from specific to general only takes into account the range from high specificity (“specific”) to low specificity (“general”). However, in colloquial language, the term “general” is typically considered to be a counterpart to the term “specific.” In this sense, specific and general are relative, so that any given measure can vary both on the level of specificity and on the level of generality. Measurements of both attitudes and behaviors can benefit from this conceptualization; even matching on single items (previously considered “specific”) can be phrased in a specific domain (e.g., “how likely are you to ride your bike to work on Monday mornings") or a general domain (e.g., “how likely are you to take alternative modes of transportation”). Thus, to clarify this literature, we consider the *domains* of both “general” and “specific.” We refer to scales that combine both domains of general and specific as “combined.” Similarly, measures of efficacy can be more specific (e.g., “to what extent do you feel that you can ride your bike to work on Monday mornings") or more general (e.g., “to what extent do you feel that you can take alternative modes of transportation”).

Based on a succinct interpretation of the literature, we define measures in the *combined* domain as involving items relevant to both specific and general domains (or, in the literature, that do not distinguish between the domains); *specific* measures as focused on a particular object or action that is typically immediate, individual, and/or direct; and *general* measures as including a broad range of objects or actions that are typically delayed, social, and/or indirect. Here, *combined* measures include pro-environmental attitudes (EA), environmental efficacy (EFF), and pro-environmental behaviors (PEBs). Measures in the *specific* domain include environmental concern (EAC), environmental self-efficacy (EFFS), and private sphere PEBs (PEBPr). Measures in the *general* domain include environmental values (EAV), environmental collective efficacy (EFFC), and public sphere PEBs (PEBPu). Respective sections below provide rationales for each of these categorizations and their relationships. Section S6 in the S0 File provides definitions and abbreviations for all relevant terms used in this text.

We label analyses *simple* if the combined measures are used, as they do not distinguish specific from general domains. We consider analyses *matched* if the relationships among attitudes, efficacy, and behavior involve all specific or all general measures. Finally, *mismatched* analyses involve relationships with a mix of specific and general measures. We raise three research questions involving these (mis)matches: (a) whether the matching of measures itself (all are specific or all are general) outperforms mismatched measures (at least one is general and at least one is specific), (b) whether the measurement domain drives stronger results (matching on specific measures out- or under-performs matching on general measures), and (c) whether either matched or mismatched measures outperform combined measures (which include all relevant specific and general measures).

We test these relationships using secondary data both across and within 11 countries. We are not proposing to test a particular environmentally-oriented macro or meso theory (a wide range of which Stern [25], and Gifford [3], summarize), but instead are only considering primary direct and moderating relationships among combined, specific, or general domains of three central components of the TPB—attitudes, control (here, efficacy), and behavior—and among matched or mismatched domains of their subscales. Nor are we proposing or testing specific differences across the countries, but instead are seeking only to identify the extent to which the overall analyses are similar across countries, and thus support more generalizability.

Thus the paper hones in on several research gaps, associated with the theory of reasoned action, in the context of environmental attitudes, efficacy, and pro-environmental behaviors, and the extent to which such concepts should be tested and implemented specifically, generally, or in combined form. The *general research question* motivated by those gaps is: How do environmental attitudes and efficacy associate with pro-environmental behaviors, considering direct or moderated models, and simple or (mis)matched models, overall or in different countries? This framing question is analyzed through direct effect moderated effect analyses of combined as well as specific or general measures, tested overall across 11 countries and 11,000 respondents, and within each of the 11 countries. Thus this study helps clarify the theoretical roles of efficacy, and specific or general measures, concerning pro-environmental behaviors across and within a large multi-country sample. The study is unique by explicitly testing two possible roles of environmental efficacy, by conceptually and empirically distinguishing specific, general, and combined measures, and by finding fairly consistent results across diverse countries.

The following sections review relevant literature on the concepts of and relationships among environmental attitudes, environmental efficacy, and pro-environmental behaviors. The next section presents the respective models (direct and moderation, simple and general, matching and mismatching) hypotheses, and a specific research question (considering results overall and by country). The methodology section describes the sample and the measures. The results section provides analytical results for each of the models, hypotheses, and specific research question. The discussion section considers interpretations of relevant results, and provides limitations and possibilities for future research, followed by a conclusion section.

## 2 Literature review

### 2.1 Environmental attitudes: Concern and values

Environmental attitudes (EA) can be defined as “a psychological tendency expressed by evaluating the natural environment with some degree of favour or disfavour” [28, p. 80]. These evaluative tendencies influence “beliefs, affects, and behaviours regarding human-environment relations” (p. 81). Gifford and Sussman [29] define EA broadly as concern for the environment or related issues. Two concepts that have shown considerable utility as indicators of EA are *environmental concern* (EAC) (more specific) and *environmental values* (EAV) (more general). In a multi-level study of secondary data from an international survey across 31 countries in 2009–2011, Tam [30] found both environmental concern and postmaterialist values and as psychological motivations associated with environmental activism engagement.

Many researchers view environmental concern (EAC) as an essential aspect of EA [31, 32]. For instance, Schultz and colleagues [33, 34] have referred to EAC as the affect associated with an EA [see also 35, p. 370], and Bamberg [36] concluded that “environmental concern” seems to be a specific part of a general environmental attitude. AlMenhali et al. [37] stated that “environmental attitudes are more of an individual concern for the physical environment, which is related to the degree of cognitive, affective, and behavioral concerns toward the environmental problems” (p. I). Environmental concern typically refers to an individual’s concern about a specific environmental condition, such as air pollution. Thus, EAC can be considered a *specific* domain of EA [38].

“Values are trans-situational goals that guide people’s evaluation of entities (e.g., person, object, social events) and selection of behaviors,” but have varying influence depending on the relevance of the value to the situation [39, pp. 1,2], among other factors. Many studies have found strong associations between environmental values (EAV) and EA [e.g., 40–42]. Some scholars have argued that EAV causally precede EA (valenced evaluations of a specific object or topic) [28, 41]. However, other researchers conceptualize EAV as one component of EA [25, 29, 43]. For example, Stern [25, p. 146] includes (perhaps inadvertently given his theory) norms, beliefs, and values as attitudinal factors influencing behaviors. Banerjee and McKeage’s [43] Environmentalism Scale has three subscales, one of which is internal environmentalism, or “attitudes about one’s own connection to nature and personally relevant issues,” similar to our values measure [29, p. 67]. Environmental values typically refers to a basic orientation toward nature or the environment in general. Thus, EAV can be considered a *general* domain of EA.

Schultz et al. [34] note that much pro-environmental attitudes research focuses on environmental values, which provide a foundation for beliefs, EAC, and PEBs. In addition, Gifford [3] includes results from several studies that demonstrate direct relationships between EAV and PEB intentions. For example, Vesely et al.’s [14] extensive meta-analyses reported a medium to strong relationship between personal connectedness to nature and pro-environmental intentions and behaviors. Chan [39] reported a positive association between self-transcendence values and PEB.

### 2.2 Pro-environmental behaviors: Private and public sphere

Pro-environmental behavior (PEB) is “behavior that consciously seeks to minimize the negative impact of one’s actions on the natural and built world (e.g., minimize resource and energy consumption, use of non-toxic substances, reduce waste production)” [19, 22, p. 240]. To some extent, environmental problems arise from the moral hazard issue and associated negative externalities: performing PEBs often requires individuals to prioritize the long-term collective health of others, a region, or the planet, over their own individual interests, while focusing on their own benefits may generate externalities such as pollution [44]. Furthermore, an individual’s PEBs are difficult to associate with larger outcomes (both perceived as well as actual) [45]. Therefore, messages promoting PEBs can highlight, or frame, benefits to either or both the individual and society [46].

Measures of PEBs are diverse, including actions that directly benefit the environment, influencing others, supporting environmental policies, and lifestyle changes. Some studies do not attempt to conceptualize distinctions among PEBs, using measures that combine different aspects of PEB [5, 10, 12, 15, 16, 20, 27, 47–50]. However, because environmental issues are public and increasingly global, PEBs involve both individual- and societal-level actions. Stern [25] distinguishes among environmental activism, nonactivist behaviors in the public sphere, private sphere environmentalism, and other (especially organizational). The third is in the private sphere, while the others are in the public sphere.

Following Milfont et al.’s [51] differentiation between public and private sphere PEBs, we identify two domains of PEBs: *private sphere pro-environmental behaviors* (PEBPr) (more specific) and *public sphere pro-environmental behaviors* (PEBPu) (more general).

Some scholars consider PEBPr as behaviors that are direct and impact-oriented (e.g., recycling) [52–54]. Other conceptualizations of PEBPr refer to the individual benefits that people accrue from performing green behaviors (termed “shallow green behavior” by Feng and Reisner [55]). However, this conceptualization does not apply equally to all private PEBs: while socially beneficial behaviors such as reducing electricity consumption can also lower an individual’s utilities cost, other behaviors such as recycling often require individuals to devote effort to separating recyclables and paying for the bin and collection, without obvious or direct individual benefit. Therefore, we define *private PEBPrs* as behaviors that single individuals can take to benefit the environment (e.g., recycling, shopping with reusable bags), and the impact of these behaviors may be direct and specific to the individual [52–54]. Thus, PEBPr is a *specific* domain of PEBs.

PEBPu are often indirect in that they can signal the intention to enact PEBs through advocating for or commitment to environmental efforts (e.g., voting for an environmental policy) [52]. Some researchers consider “influencing others” as a separate facet of PEB [56]; based on the above conceptualization, however, influencing others can be considered an indirect and a public sphere behavior. Piyapong [53] further distinguishes public sphere (intent-oriented) behaviors into activist and non-activist. Homburg and Stolberg [57] consider nonactivist behaviors as a form of social commitment, such as engaging in environmental protection actions. Chen [58] measures public nonactivist behavior as “specific social commitments” such as planting trees and picking up litter on the beach (p. 70). We define PEBPu as behaviors that require some kind of group organization to benefit the environment either directly (e.g., volunteering to plant trees) or indirectly (e.g., signing a petition to support an environmental cause). Further, the impact of these behaviors may be diffuse and collective, requiring other engaged individuals [e.g., 52]. Thus, PEBPu is a *general* domain of PEBs.

### 2.3 Efficacy: Self and collective

Scholars have pointed to the role of efficacy (EFF) in enabling or motivating individuals to translate attitudes into concrete action [19]. EFF, or the belief that one has capabilities to “organize and execute the courses of action required to produce given attainments,” allows individuals to feel that their actions are worthwhile and achievable [18, p. 3]. EFF both “motivates and sustains” behavior change [59, p. 2] by focusing attention [60], affecting perception of goal difficulty and goal commitment [61], helping assign resources to the goal [62], and fostering searching for better strategies [63, 64; see 64, pp. 660–661 for a review].

Researchers have conceptualized EFF in both *specific* and *general* forms. Wang and Richarde [65] concluded that task-specific and global measures of EFF were relatively distinct. In the context of work motivation, Eden [66] referred to the concept of “total subjective efficacy” as including both specific (e.g., tools, supervisory leadership) and general (e.g., the organization or team) subjective efficacy. Schwarzer [67] validated across 14 cultures and 13 languages a measure of generalized self-efficacy (ability to manage a variety of stressors) that differentiated task-specific from general efficacy. This scale was further validated in three and then in five countries and languages [68, 69, respectively].

Other studies have reported varying relationships between self-efficacy and general efficacy. For example, Hanss and Böhm [56] concluded that dimensions of sustainable development (domain-specific) self-efficacy were variously associated with three kinds of sustainable behavior, while general self-efficacy was not. Smith et al. [70] also separately measured task-specific and general self-efficacy, showing that engaging in an unsolvable problem negatively affected the former but not the latter.

We refer to the concept as *environmental efficacy*, the combined scale as *EFF*, and the two domains as *self-efficacy* (EFFS) (more specific) and *collective efficacy* (EFFC) (more general). In the context of explaining PEBs, distinguishing between EFFS and EFFC is especially relevant because individual actions are insufficient for most environmental problems. For example, Hamann and Reese [71] reported that EFFS predicted PEBPr, although the relationship between EFFC and PEBPu remained unclear. Section S1 in the S0 File provides an extended review and justification of self- and collective efficacy.

EFFS typically consists of two aspects: whether the person believes that (a) that they can perform a given action, and (b) that the given action will have the intended effect [18, 64, 71]. EFFS also includes the ability to overcome some barriers in performing a behavior [9], and is conceptually similar to perceived behavioral control in the TPB [9, 17, 72]. Generally considered domain-specific [18], EFFS strengthens motivation and behavioral intentions [27, 47, 64]. EFFS, through increasing a sense of empowerment and effectiveness, should motivate relevant environmental behavior [72]. We note that, like PEBPr, EFFS is specific to the individual [18]. Therefore, we consider EFFS a *specific* domain of EFF.

Bandura [18] conceptualized EFFC as “a group’s shared belief in its conjoint capabilities to organize and execute the courses of action required to produce given levels of attainments” (p. 477). Collective efficacy also includes two aspects: (a) whether a group believes that they collectively can perform a certain behavior, and (b) whether the behavior has the desired effect. Perceived collective efficacy can allow individuals to believe that group efforts may matter even though individual efforts are insufficient [73]; see also the social identity model of collective action [74]. Chen [58] further clarifies that collective efficacy is an “emergent group-level property and not merely the sum of the efficacy beliefs of the individual members” (p. 69), and that the group must rely on collective rather than individual resources [see also 75]. For sustainable development issues, Hanss and Böhm [56] found that “in this country” and “around the world” did not represent different facets of geographical collectivity. Similar to PEBPu, EFFC is general in the sense that it involves an assessment of the group rather than of the individual [18]. Therefore, EFFC is a *general* domain of efficacy.

## 3 Relationships among EA, EFF, and PEBs—Simple direct or moderation effects, (mis)match direct or moderation effects—With controls/covariates and by country; models, hypotheses, and research question

### 3.1 Simple model (direct and moderation; ignoring specific-general distinctions)

We have already noted the familiar direct EA-PEBs model, and we use that as the basic model to which we add EFF. EFF could play at least three roles related to PEBs: as a direct association with behaviors, as a moderator of the attitude-behavior relationship, or as a mediator between attitudes and behaviors. Section S2 in the S0 File discusses why this study does not consider the mediation role. As a central goal of this study is to clarify the relationships between these constructs, we will test both the direct role of EFF and the less-examined moderation role of EFF in the EA-PEB relationships. Thus, the direct and moderation models will first be tested using the three combined concepts (EA, EFF, PEBs; we refer to these as *simple* models), and then the direct and moderation models will be tested using the combinations of matches and mismatches of the concepts’ domain subscales (we refer to these as *[mis]match* models, discussed in Section 3.2).

Studies consistently find that higher levels of EFF are directly associated with more engagement in a wide range of PEBs [e.g., 47, 58, 71, 76]. This direct effect approach is grounded in the TPB [12, 15, 77, 78], social cognitive theory (SCT) [71, 79], and protection motivation theory (PMT) [9, 78].

There are far fewer studies proposing or testing a moderation effect of efficacy. Although several studies do not find a moderating role of self-efficacy on the relationship between attitudes and PEBs [7, 11, 24, 45, 80], a few studies have shown significant moderation effects. For example, Berger and Corbin [20] demonstrated that both individuals’ own perceived consumer effectiveness (EFFS) and their faith in the efficacy of others (EFFC) moderated the relationship between participants’ EA and environmentally responsible purchasing behaviors (PEBs).

### 3.2 (Mis)matching models (direct and moderation, including specific-general distinctions)

One implication of the reviews in Section 2 is that more subtle approaches to the relationships among EA, EFF, and PEBs would take into account the specific and general domains within EA (i.e., EAC, EAV), within EFF (EFFS, EFFC), and within PEBs (PEBPr, PEBPu). Thus, more domain-specific EAs are more likely to be related to more domain-specific PEBs, more domain-general EAs are more likely to be related to more domain-general PEBs, and EFF domains might also be more effective, either directly or as a moderator, when matched with specific or general domains of EA and PEBs [81]. Nonetheless, even mismatched domains are likely to be significantly associated; for example, a generalized conservation attitude may be associated with multiple specific forms of conservation [29].

### 3.3 Controls

Both the simple and (mis)match models will control for relevant demographics. Research has identified a wide variety of other influences, here conceptualized as control variables, on environmental attitudes and behaviors. Among others, these include age, gender, education, geographic location, income, social class, and environmental social norms [9, 19, 53, 82, 83]. For example, Gifford [3] notes that rural and urban residents have different knowledge of and experience with environmental issues, place attachment, and beliefs in nature as a consumption resource or as deserving preservation for its own sake. Studies have found differences in concerns about and attitudes toward environmental problems across the rural-urban continuum [84], though other results are mixed [3, 29].

### 3.4 A multi-country perspective

Although the basic relationships among EA, EFF, and PEBs may be an enduring framework, the levels and relationships among these constructs may also differ somewhat across countries. For example, in Brazil and Australia, deep histories of environmentalism have led voters to prioritize the environment as a central political issue, while in Indonesia, the multi-billion-dollar palm oil industry has led to incentivized deforestation [85, 86]. Variations in countries’ infrastructure, policies, assumptions, ideology, cultural history, and economic availability regarding environmental issues can all influence individuals’ attitudes and abilities to translate those attitudes into action.

Tam and Milfont [87] summarized characteristics of 54 cross-cultural environmental articles in the *Journal of Environmental Psychology* from 2000–2019. A number of studies examine EA and PEBs outside of the United States. Many studies involve samples from one or two countries [e.g., 27, 57, 88]. For example, Kim et al. [9] concluded that EFFS was a stronger predictor of PEBs among American compared to Korean participants. In addition, several projects do consider large numbers of countries. Gifford and Sussman [29] provide a succinct review of similarities and differences in EA and EAC across a number of cross-national studies (p. 69); Chan [39] noted some prior cross-country studies on values-PEB relationships; Schultz et al. [34] found consistent associations between EAV and EAC across six countries; Wang’s [13] study of EAs and sustainable consumption behaviors in 31 countries found that in low-income countries, individual attitudes are stronger predictors of sustainable behaviors under high levels of environmental governance but weaker when environmental governance is lacking; and Oreg and Katz-Gerro [12] ran a multilevel model on a very large sample across 27 countries, showing that country-level postmaterialism values influenced participants’ level of EA, which then predicted their PEBs. Beyond the central goal of this study to test simple and (mis)matched, direct and moderation, relationships among both scales and subscales of EA, EFF, and PEBs, we also provide a global perspective from 11 diverse countries, describing similarities and differences in the relationships.

However, this study does not propose or test hypotheses about country-level differences, as it uses only individual-level data (see 6.2 Limitations and Future Directions, and Sections S4 and S5 in the S0 File). We apply the method of cross-cultural replication, comparing patterns of results across countries [87], though not via statistical tests. We refer to the cross-country sample as *overall* and the separate within-country sample as *by country*.

### 3.5 Models, hypotheses, and research questions relating EA, EFF, and PEBs, via direct and moderation effects, simple and (mis)matched models, overall and by country

**Fig 1:**
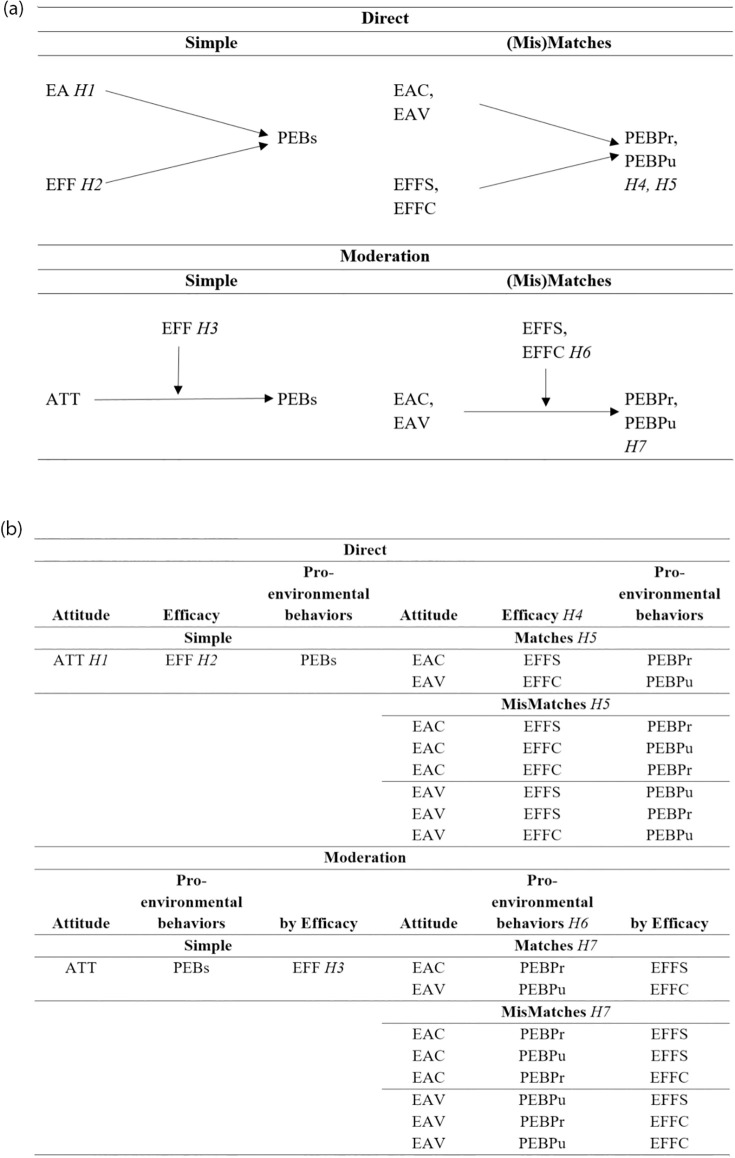
Visual and tabular summary of models: Direct or moderation, simple or (Mis)matches. **A. Visual Portrayal of Models. B. Textual Portrayal of Models**. [Legend at bottom of B] Note: Hypotheses are indicated by italics. All models are tested overall and by country: *RQ1* ATT: Environmental attitudes; EAC: Environmental concern; EAV: Environmental value; PEB: Pro-environmental behavior; PEBPr: Private Pro-environmental behavior; PEBPu: Public Pro-environmental behavior; EFF: Efficacy; EFFS: Environmenal self-efficacy; EFFC: Environmental collective efficacy.

Based on the above reviews, we propose a general model whereby EA relates positively to PEB (e.g., [30]), and EFF relates positively to PEB directly (e.g., [47, 58, 71, 76]) and as a moderator in the EA-PEB relationship (e.g., [7, 11, 24, 45, 80]). Given previous research that has found significant relationships between these constructs across a range of countries (e.g., [9, 12]), we expect this general model to be significant overall (across countries). Fig 1 portrays all the direct and moderation models, and the combined and (mis)matched models, in both visual and tabular form, and indicates the following hypotheses and research question.

The concepts used in each model are as follows. The simple overall model includes EA, EFF, PEBs. The two matching models include: 1: EAC, EFFS, and PEBPr; and 2: EAV, EFFC, and PEBPu. The six mismatching models include: 3: EAV, EFFS, and PEBPr; 4: EAC, EFFC, and PEBPu; 5: EAV, EFFS, and PEBPu; 6: EAV, EFFC, and PEBPr; 7: EAC, EFFS, and PEBPu; and 8: EAC, EFFC, and PEBPr. In addition to direct effects for each of those models, the moderation effect model includes the interaction between EA, EAC, and EAV, with EFF, EFFS, and EFFC, respectively.

First, we propose two simple direct effects.

**H1:** EA will be positively related to PEBs.
**H2:** EFF will be positively related to PEBs.

Next, we propose a simple moderation effect.

**H3:** The positive relationship between EA and PEBs will be positively moderated by EFF.

Then we test for the more subtle direct effects by considering two matches and six mismatches of the domains of EA, EFF and PEBs. As in the traditional TPB model, all listed (mis)match relationships should be positive [17], so we do not propose specific hypotheses for each, but rather group them under H4. However, based on the discussions in Section 2, we would expect that matches should explain more variance in PEBs than the mismatches (e.g., [15]), thus H5.

**H4:** All combinations of EA subscales and of EFF subscales are positively related to all combinations of PEB subscales.
**H5:** The two matched direct relationships (model 1: EAC, EFFS, PEBPr; model 2: EAV, EFFC, PEBPu) will explain more variance in PEBs than each of the six mismatched direct relationships (models 3–8).

Following, we test for more subtle moderation effects by considering the two matches and six mismatches of EA, EFF and PEBs. For example, the relationship of EAV with PEBPu moderated by EFFC should be positive (e.g., [17]); and explain more variance than when moderated by EFFS (e.g., [15]).

**H6**: All relationships of combinations of EA domains (EAC, EAV) to all combinations of PEB domains (PEBPr, PEBPu) are positively moderated by all combinations of efficacy domains (EFFS, EFFC).
**H7**: The two matched moderation models (EAC*EFFS, PEBPr; EAV*EFFC, PEBPu) will explain more variance in PEBs than each of the six mismatched moderation relationships.

For the separate country analyses, for parsimony, we use only (a) the simple direct and moderation effects models, and (b) the (mis)matched model in the overall analyses explaining the most variance.

**RQ1**: In what ways are the results from the simple direct or moderation effects model, and the (mis)matched model that explains the most variance, similar or different across 11 countries?

## 4 Methodology

### 4.1 Sample

The data are responses from 1,000 adults 18 years or older in each of 11 countries (United States, Mexico, Brazil, United Kingdom, South Africa, Kenya, China, South Korea, Australia, United Arab Emirates, and Indonesia; total N = 11,000) to a survey conducted in January and February 2019 by Ipsos for the National Geographic Society (NGS). They followed their standard practices, and later provided the data to the researchers. The data are thus secondary, as well as anonymous, and we had no role in obtaining consent, so the authors did not need to obtain IRB review. Further, we use only a small set of the wide range of measures from the survey. The countries reflected the NGS’s initiatives at the time, focused on reducing humans’ environmental footprint. The sample sizes were selected to achieve an approximately +/-5% margin of error at the country level. The large country sample sizes have a power value of 1.00 for an estimated effect size of .15. All surveys were conducted online (Ipsos reports the samples are representative of Internet users in those countries), except for Kenya, which were obtained via computer-aided face-to-face interviews. Interviews were conducted in English, the country’s native language, or English and multiple languages in South Africa and Kenya. Age and gender quotas were applied to reflect census data, so the data are not weighted. Items within each question were randomized.

After the data were collected, the authors were invited by NGS to collaborate in the analysis and publication of findings from these data. Tam and Milfont [87] summarize three recruitment methods for multi-country studies, including (a) convenience sampling, (b) contracting research companies (typically applying quota sampling), or (c) using existing international datasets and their relevant survey questions. We used method (c), which collected data via method (b). Thus, our study is a secondary analysis, involving typical strengths and weaknesses of measures and data from a prior project. In particular, we emphasize that (a) all the items used in the analyses are selected from a prior dataset which we did not design, so more standard or valid measures were not available; (b) we do not have access to their research or literature justification for the specific items or specific countries analyzed, except as noted; and (c) we created combined scales and subscales from available items that corresponded to our notions of more specific and more general domains, so the subscales do not represent the most valid or explicit operationalizations. This study is therefore opportunistic, exploratory, and limited, yet extends the simple direct effects model, and tests it across large samples in 11 countries.

### 4.2 Measures

A variety of methodological issues attend such a study using non-standard measures, multiple concepts, direct and moderation analyses, and large sample sizes across multiple countries. Sections S1–S5 in the S0 File provide in-depth results and/or discussions concerning the absence of mediation analyses, exploratory factor analysis loadings of separate and all subscale items, confirmatory factor analysis loadings and fit for separate subscales and scales, reliabilities, discriminant validity, scale factor loadings and congruence across countries, country-level effects in the overall analyses, and statistical differences between models and countries. This section describes each measure used in the models, provides sample items, and the scale Cronbach’s α. S3.1 Table in S1 File provides the wording for the items measuring EA, EFF, and PEBs.

*Environmental attitudes*. *EAC* was measured by asking participants to indicate their level of concern for eight global issues including “habitat loss” and “lack of clean drinking water,” with response options ranging from 1 (*not at all concerned*) to 5 (*very concerned*). After dropping two items, α = .88. *EAV* was assessed through six items from the Moral Conviction Scale & Values Scale [89], including “Nature has its own value, independent of its value to people” and “Nature is important to me, to who I am as a person,” with response options ranging from 1 (*strongly disagree*) to 5 (*strongly agree*). After removing one item, Cronbach’s α = .84. A combined measure of *pro-environmental attitudes* (EA) was created by taking the mean of the mean of the EAV scale and the mean of the EAC scale (because of the different number of items in each, 5 and 6 respectively). Cronbach’s α for the 11-item scale is .90.

*Pro-environmental behaviors*. Participants were asked to indicate how frequently they personally engaged in six PEBs over the past 12 months, such as “recycle” and “talk to friends or family about an environmental issue,” with response options ranging from 1 (*never*) to 5 (*all the time*). Kaiser [23] notes that “…there is no agreement about which behavior domains can be aggregated. A common way of aggregation is an empirical one,” such as by factor analysis (p. 397). Thus, the principal component analysis of PEBs demonstrated that three of five items loaded onto one factor (*PEBPu*), while the remaining two loaded onto another factor (*PEBPr*). One item did not load cleanly onto either component and was removed.

The two-item PEBPr scale (recycle, reusable bags) had a low alpha and Spearman-Brown coefficient of .60 [90]. However, a lower α for instruments designed to measure multifaceted constructs is often expected, especially when limited to a low number of question items [91], so such a measure can still be highly useful with a low α. The three-item PEBPu scale had an α of .76. A combined measure of PEBs was created by taking the mean of the means of PEBPr and PEBPu (as the two measures had unequal numbers of items). The combined five-item scale had an α of .71.

*Environmental efficacy*. Participants’ level of EFFS was measured by asking participants to “Please rate how confident you are that YOU AS AN INDIVIDUAL can attain the following goals in the next 10 years,” with four items including “protect habitats” and “save animals at risk of extinction,” and response options ranging from 0 (*cannot do at all*), 50 (*moderately can do*), to 100 (*highly certain can do*; α = .85). Level of EFFC was measured by asking participants to “Please rate how confident you are that YOUR COUNTRY can collectively attain the following goals in the next 10 years,” with the same items and response scale used to measure EFFS (α = .89). A combined measure of EFF was created by taking the mean of participants’ scores on the eight EFFS and EFFC measures (α = .89).

*Control variables*. Participants reported their *age* in years. Participants indicated their gender as 1 (Male), 2 (Female), 3 (Other), or 4 (Prefer not to say). As less than .2% reported the last two, those were dropped from analyses, and gender was recoded as 0 (Male) and 1 (Female). Participants indicated their *residential location* by whether they currently live in a 1 (*rural*), 2 (*suburban*), or 3 (*urban*) area. *Socio-economic ladder* (*SES Ladder*) was measured by asking participants to respond to the adapted question 2 from the MacArthur Scale of Subjective Social Status [92]. This item included a picture of a 10-rung ladder ranging from 1 (*at bottom*) to 10 (*at top*) and stated, “The ladder below represents where people stand in your country’s society. At the top of the ladder are the people who are the best off, those who have the most money, most education, and best jobs. At the bottom are the people who are the worst off, those who have the least money, least education, worst jobs, or no job. Please select the rung that best represents where you think you stand on the ladder.” Participants reported their *education* level by responding to, “Which of the following comes closest to the last level of education you completed?” with various categories appropriate to the country. Because of the wide variation in this measure across countries, the results were standardized within each country to enable comparison across countries. Participants estimated *descriptive environmental social norms* by answering the question, “What percentage of people do you think engage in environmentally friendly behaviors, such as buying recycled, organic, or biodegradable products or saving energy in your country?” by entering a percentage from 0 to 100.

### 4.3 Analyses

After data collection, measure development, and data dimensionality, reliability, and validity, discussed above, we then conducted a series of analyses. First were basic descriptive statistics, and then correlations among the combined, specific, and general measures. Next were hierarchical regressions using the full dataset for each of the simple direct and moderation effects models, with combined, specific, and general measures, organized into match models and mismatch models. For country-level analyses, we used the simple direct effects and moderation effects, and for parsimony, the one (mis)match model explaining the most variance in the overall analyses. Results from each of labeled according to the respective hypotheses or the research question.

## 5 Results

### 5.1 Overall (across all countries)

Table 1 presents descriptive statistics overall and for each country, for the combined scales and their specific/general subscales.

**Table 1:** Descriptives for scales and subscales, overall and by country.

Model Variables	All	US	Mex	Bra	UK	SA	Ken	Chi	SK	Aus	UAE	Indo
EAC: Environmental concern (6 concern items)	4.27/ .73	4.01/ .86	4.59/ .56	4.50/ .63	4.09/ .74	4.47/ .63	4.33/ .76	4.13/ .62	4.18/ .68	4.11/ .76	4.11/ .84	4.48/ .62
EAV: Environmental values (5 value items)	4.29/ .68	4.11/ .75	4.55/ .55	4.43/ .59	4.11/ .70	4.42/ .60	4.40/ .68	4.33/ .55	3.99/ .62	4.07/ .75	4.27/ .79	4.56/ .54
EA: Pro-environmental attitudes (mean of the mean of 6 Concern items and 5 Values items)	4.28/ .63	4.06/ .72	4.57/ .49	4.47/ .54	4.10/ .65	4.44/ .55	4.36/ .61	4.23/ .51	4.08/ .58	4.10/ .68	4.19/ .73	4.52/ .49
EFFS: Self-efficacy index (4 items)	47.31/ 26.10	38.93/ 26.92	57.05/ 26.68	55.83/ 27.34	37.32/ 24.56	46.79/ 26.39	47.42/ 22.65	50.77/ 23.68	42.55/ 22.41	41.10/ 26.68	48.60/ 25.59	54.06/ 24.84
EFFC: Collective efficacy index (4 items)	47.87/ 25.23	46.07/ 26.40	50.39/ 26.52	47.33/ 28.63	44.39/ 24.36	40.73/ 26.94	52.70/ 20.80	49.78/ 22.79	43.38/ 21.16	47.61/ 25.79	52.17/ 25.78	52.03/ 24.12
EFF: Efficacy (mean of 4 Self- & 4 Collective efficacy items)	47.59/ 22.74	42.50/ 23.57	53.72/ 23.34	51.58/ 24.28	40.85/ 21.34	43.76/ 22.66	50.06/ 18.22	50.28/ 21.89	42.97/ 19.65	44.35/ 23.34	50.39/ 23.71	53.05/ 22.53
PEBPr: (2 private PEB items)	3.90/ .91	3.71/ 1.08	3.95/ .81	3.77/ .91	4.57/ .65	3.81/ .96	3.58/ .86	3.98/ .68	3.88/ .82	4.45/ .69	3.79/ .95	3.46/ .88
PEBPu: (3 public PEB items)	3.26/ .95	2.68/ .99	3.58/ .81	3.46/ .87	2.81/ .92	3.31/ .94	3.13/ .92	3.60/ .71	3.03/ .83	2.87/ .98	3.73/ .89	3.66/ .76
PEBs (mean of mean of 2 PEBPr and mean of 3 PEBPu)	3.58/ .76	3.19/ .88	3.77/ .71	3.61/ .79	3.69/ .58	3.56/ .84	3.35/ .74	3.79/ .59	3.45/ .70	3.66/ .63	3.76/ .83	3.56/ .73
**Demographics**												
Age (Years)	41.14/ 15.28	48.62/ 18.61	40.13/ 14.36	41.35/ 14.30	47.66/ 17.45	37.67/ 13.97	34.00/ 12.18	41.11/ 13.59	44.40/ 14.17	45.66/ 15.86	33.65/ 9.89	38.26/ 13.20
Gender												
0 (Male)	52.0%	48.1	48.1	47.8	49.7	47.3	49.2	50.9	49.5	48.7	72.9	50.2
1 (Female)	48.0	51.2	51.3	52.2	50.0	52.7	50.8	49.1	50.5	50.9	27.1	49.8
Location	2.48/ .70	1.96/ .73	2.78/ .52	2.87/ .43	2.03/ .71	2.26/ .71	2.61/ .66	2.88/ .36	2.79/ .53	2.04/ .59	2.47/ .77	2.55/ .65
1 (Rural)	12.1%	28.6	5.0	3.5	24.0	15.7	9.6	1.1	5.9	15.7	17.2	8.4
2 (Suburban)	26.8	47.3	12.5	6.3	49.1	42.8	19.9	10.0	8.9	65.1	18.2	27.7
3 (Urban)	61.1	24.1	82.5	90.2	26.9	41.5	70.5	88.9	85.2	19.2	64.6	63.9
SES ladder	5.26/ 1.94	5.67/ 1.97	4.75/ 1.53	5.35/ 1.76	5.92/ 1.90	5.63/ 1.87	5.77/ 1.85	5.18/ 1.61	5.89/ 1.84	5.70/ 2.02	3.53/ 1.82	4.49/ 1.63
Education (Z-score)	.00/ 1.00	.00/ 1.00	.00/ 1.00	.00/ 1.00	.00/ 1.00	.00/ 1.00	.00/ 1.00	.00/ 1.00	.00/ 1.00	.00/ 1.00	.00/ 1.00	.00/ 1.00
Environ. soc. norms (%)	40.06/ 22.51	39.61/ 21.01	33.17/ 19.44	33.98/ 21.69	42.71/ 21.84	33.74/ 21.23	43.19/ 22.29	42.40/ 23.75	35.88/ 21.62	45.88/ 22.53	47.73/ 23.56	42.32/ 22.48

N = 11,000 overall; 1,000 per country; Note: values are M/SD, except % for Gender.

As Table 2 shows, all bivariate correlations among the combined scales and their subscales were positive and significant (somewhat due to large sample size). Notably, correlations of EA with PEBs were stronger (.22-.43) than were the correlations of EA with EFF (.08-.32), and with PEBPu (.15-.43) than with PEBPr (.08-.25).

**Table 2:** Correlations among scales and subscales, overall.

**Model Variables**	**EAC**	**EAV**	**EA**	**EFFS**	**EFFC**	**EFF**	**PEBPr**	**PEBPu**
EAC	--							
EAV	.58	--						
EA	.90	.88	--					
EFFS	.22	.24	.26	--				
EFFC	.08	.13	.12	.57	--			
EFF	.17	.21	.21	.89	.88	--		
PEBPr	.22	.23	.25	.12	.08	.11	--	
PEBPu	.37	.40	.43	.32	.15	.27	.32	--
PEBs	.37	.39	.42	.27	.14	.24	.80	.82

N = 11,000

Off-diagonal values are Pearson correlations; all *p* < .001, 1-tailed

In the overall model (across all countries), to explore the unique variance explained by each variable, and to avoid non-robust cluster errors, we use hierarchical multiple regressions with dummy-coded countries to test both the simple and the (mis)matching models, predicting combined PEBs and its two subscales, respectively. Dummy-coded country variables, with the U.S. as the reference group (using the GLM default settings), were entered in block 1, and EA and EFF variables along with their respective centered interaction term were entered in block 2. Age, gender, location, SES ladder, education, and environmental social norms (descriptive) were entered in block 3, to assess the explanatory strength of the central model variables before indicating the additional variance explained by the demographic controls.

Table 3 presents results from the simple model and the eight (mis)matching models. All models were significant at *p* < .001. The simple model, using EA, EFF, and PEBs, the interaction between EA and EFF, and demographics, explained 31% of the variance in PEBs. Dummy-coded country variables explained 6.0% of the variance in PEBs. Both EA and EFF explained significant variance (supporting H1 and H2, respectively), although the interaction of EA and EFF was not (rejecting H3); together, they explained 27% of the variance in PEBs. Finally, age, gender, SES ladder (negatively), education, and environmental social norms (descriptive) were all significant, while residential location was not. Together, the demographics explained 4% of the variance in PEBs.

**Table 3:** Hierarchical regressions for simple direct and moderation effects models using combined scales, and using (Mis)match subscales, overall.

Model Variables	Simple Model	Match Models (1, 2)		MisMatch Models (3–8)					
	EA/ EFF/ PEBs	1: EAC/ EFFS/ PEBPr	2: EAV/ EFFC/PEBPu	3: EAV/EFFS /PEBPr	4: EAC/ EFFC/PEBPu	5: EAV/EFFS /PEBPu	6: EAV/ EFFC/PEBPr	7: EAC/ EFFS /PEBPu	8: EAC/ EFFC/ PEBPr
**Country** a										
Mex	.12***	.01	.20***	.02	.18***	.17***	.02*	.16***	.02
Bra	.10***	-.04***	.19***	-.03**	.17***	.16***	-.02	.15***	-.02**
UK	.16***	.27***	.04***	.27***	.03***	.04***	.27***	.04***	.26***
SA	.10***	.01	.15***	.02	.14***	.14***	.02	.13***	.02
Ken	.02	-.06***	.08***	-.06***	.08***	.07***	-.07***	.07***	-.05***
Chi	.19***	.06***	.23***	.05**	.24***	.21***	.05**	.22***	.07***
SK	.11***	.04**	.13***	.07***	.10***	.12***	.06***	.09***	.04**
Aus	.14***	.22***	.05***	.23***	.03***	.05***	.23***	.03**	.22***
UAE	.17***	.02	.23***	.01	.23***	.22***	.01	.23***	.02
Indo	.06***	-.14***	.20***	-.14***	.19***	.18***	-.14***	.18***	-.13***
***Adj R*** ^***2***^	.06	.12	.14	.12	.14	.14	.12	.14	.12
***F*(10, 10966)**	75.87***	155.18***	181.62***	155.18***	181.62***	181.62***	155.18***	181.62***	155.18***
**Attitude & Efficacy**										
EAC	--	.26***	--	--	.33***	--	--	.30***	.27***
EAV	--	--	.33***	.26***	--	.30***	.28***	--	--
EA	.41***	--	--	--	--	--	--	--	--
EFFS	--	.10***	--	.10***	--	.16***	--	.16***	--
EFFC	--	--	.04***	--	.05***	--	.04***	--	.05***
EFF	.11***	--	--	--	--	--	--	--	--
Inter EAx*EFFx b	-.004	.01	-.01	-.01	-.02*	-.02**	-.02*	-.01	-.01
***Adj R*** ^***2***^	.27	.21	.26	.22	.26	.29	.21	.29	.20
***F*(13, 10963)**	311.56***	228.69***	299.20***	241.53***	292.23***	346.90***	229.56***	337.22***	216.89***
**Demographics**										
Age	.04***	.14***	-.04***	.13***	-.03***	-.04***	.12***	-.03*	.14***
Gender	.04***	.06***	.03**	.06***	.02*	.03***	.06***	.02*	.05***
Location	.01	.04***	-.00	.03***	.00	.00	.03**	.00	.04***
SES ladder	-.14***	-.05***	-.15***	-.04***	-.16***	-.14***	-.05***	-.15***	-.06***
Education (Z-score)	.05***	.04***	.06***	.04***	.06***	.06***	.04***	.06***	.04***
Environ. soc. norms	.13***	.10***	.14***	.09***	.14***	.11***	.10***	.12***	.11***
***Adj R*** ^***2***^	.31	.25	.31	.25	.31	.33	.24	.33	.24
***F*(19, 10957)**	106.54***	189.79***	260.03***	194.94***	260.334***	286.44***	187.31***	284.98***	182.25***

N = 11,000

For comparability, values are *β* coefficients; tables of B coefficients, SEs, and CIs are available from the corresponding author.

* *p* < .05;

** *p* < .01;

*** *p* < .001

^a^. US is the reference country for the dummy codes.

^b^. The appropriate centered environmental attitudes (x) and efficacy (x) terms were used to compute the respective interaction terms.

Tests of the (mis)match models highlight small differences in relationships among subscales of EA, EFF, and PEBs. Across all matching (1–2) and mismatched models (3–8), EFFS (*β* from .10 to .16) was more influential than EFFC (*β* from .04 to .05). In addition, PEBPr compared to PEBPu involved (a) less overall variance explained (adj R^2^ from 24 to 25% compared to from 31 to 33%), and (b) slightly weaker effects of EAC and EAV (*β* from .26 to .28 compared to .30 to .33). Concerning demographics, PEBPr compared to PEBPu was associated with (c) stronger effects of age (also positive, compared to negative associations for PEBPr), (d) very slightly stronger effects for females, (e) stronger positive effects of residential location (compared to none), (f) substantially weaker negative effects of the SES ladder, (g) very slightly weaker positive effects of education, and (h) slightly weaker effects of environmental social norms.

The role of efficacy as a moderator hardly mattered or varied throughout the (mis)match models (*β* from -.02 to .01). In three of the (mis)match models (4, 5, and 6), the EA-EFF interaction was barely significant; in all other models, it was not significant. Surprisingly, in all of those, the slight moderation by efficacy (whether self- or collective) was negative (e.g., *β* = -.02). EFFS significantly negatively moderated the relationship between EAV and PEBPu (model 5), while EFFC significantly negatively moderated the relationship between EAC and PEBPu (model 4), and between EAV and PEBPr (model 6).

The (mis)match models that explained the greatest amount of variance were, unexpectedly, models 5 and 7, including EAV, EFFS, and PEBPu (adj R^2^ = .33, *F* = 286.44, *p* < .001; model 5), and EAC, EFFS, and PEBPu (adj R^2^ = .33, *F* = 284.98, *p* < .001; model 7). Although we expected the two matching models (1, 2) to explain more variance than the mismatch models (3–8), the stronger results for these two mismatch models align with the findings that PEBPu models have higher adj R^2^ values than PEBPr models, and that EFFS has higher *β*s than EFFC. For parsimony, our subsequent analyses consider only the first mismatch model 5.

### 5.2 By country

For the country-specific models, the combined and the subscale measures (by domain) of EA and EFF were entered in block 1, along with the product of their within-country centered terms as the interaction term. Demographics were entered in block 2.

Hierarchical regressions were computed for each country for both the simple models (Table 4) and for the one mismatch model explaining the most variance (i.e., model 5 in Tables 4 and 5). At the country level, a visual inspection of results from both simple and mismatch models indicates that the simple model explained the same or more total variance in PEBs than mismatch model 5 in all countries except Kenya and Indonesia. Furthermore, in every country besides Indonesia, EA had a stronger association with PEBs in the simple model (*β* from .26 to .55) than did EAV in the mismatch model 5 (*β* from .05 to .42). Still, EFFS was slightly more strongly associated with PEBs in the mismatch model (*β* from .04 to .22) than was EFF in the simple model (*β* from .03 to .18) in every country besides China.

**Table 4:** Hierarchical analysis for simple model, by country.

Model Variables	US	Mex	Bra	UK	SA	Ken	Chi	SK	Aus	UAE	Indo
**Attitude & Efficacy**											
EA	.48***	.33***	.26***	.54***	.36***	.16***	.46***	.44***	.53***	.55***	.33***
EFF	.12***	.16***	.18***	.09**	.15***	.06	.06*	.12***	.06**	.03	.16***
Inter EA*EFF a	.03	-.03	-.03	.01	-.08**	-.02	-.03	.05	.03	.03	-.01
***Adj R*** ^***2***^	.31	.18	.17	.34	.20	.03	.24	.28	.33	.31	.18
***F***	(3, 986) 150.94***	(3, 990) 75.76***	(3,996) 68.73 ***	(3,993) 172.88***	(3,996) 85.07***	(3,996) 12.76***	(3,996) 105.99***	(3,996) 128.44***	(3,992) 167.32***	(3,996) 147.85***	(3,996) 71.48***
**Demo-graphics**											
Age	-.01	-.02	.11***	-.02	.07*	.02	.03	.06*	.01	.04	.05*
Gender	.06*	.09**	.01	.06*	.01	.01	.04	.03	.04	.01	.03
Location	.06*	-.01	.05	.02	-.03	-.15***	.09***	.09***	-.04	-.00	.06*
SES ladder	-.12***	-.11***	-.07*	-.11***	-.14***	-.04	-.23***	-.13***	-.13***	-.21***	-.11***
Education	.10***	.06	.10***	.07*	.02	-.07*	.05*	-.01	.09**	.05*	.18***
Environ. soc. norms	.18***	.13***	.15***	.09***	.16***	.07*	.09**	.19***	.13***	.17***	.14***
***Adj R*** ^***2***^	.38	.23	.23	.37	.25	.07	.32	.34	.38	.39	.26
***F***	(9, 980) 67.60***	(9, 984) 33.02***	(9, 990) 33.55***	(,987) 66.29***	(9,990) 37.25***	(9,990) 8.67***	(9,990) 53.54***	(9,990) 57.82***	(9,986) 68.79***	(9,990) 72.16***	(9,990) 40.19***

N ~ 1,000 per country

Values are *β* coefficients; table of B coefficients, SEs, and CIs are available from the corresponding author.

* *p* < .05;

** *p* < .01;

*** *p* < .001

^a^. The appropriate within-country centered environmental attitudes and efficacy terms were used to compute the respective interaction term.

**Table 5:** Hierarchical regressions for model 5 subscale mismatch (values/self/public), by country.

Model Variables	US	Mex	Bra	UK	SA	Ken	Chi	SK	Aus	UAE	Indo
**Attitude & Efficacy**											
EAV	.37***	.25***	.22***	.40***	.30***	.05	.37***	.31***	.40***	.42***	.33***
EFFS	.19***	.20***	.22***	.18***	.19***	.14***	.04	.17***	.16***	.10***	.19***
Inter EAV*EFFS a	.02	-.02	-.11***	-.03	-.05	-.02	.01	.02	.02	-.03	-.00
***Adj R*** ^***2***^	.26	.14	.17	.23	.17	.04	.16	.19	.25	.21	.19
***F***	(3,986) 115.26***	(3,990) 55.89***	(3,996) 67.50***	(3,993) 100.07***	(3,996) 69.23***	(3,996) 14.83***	(3,996) 62.94***	(3,996) 77.94***	(3,992) 114.16***	(3,996) 90.05***	(3,996) 77.84***
**Demographics**											
Age	-.17***	-.04	.07**	-.15***	.01	-.02	-.01	.02	-.11***	.04	.05
Gender	.04	.08**	.01	.05	.02	.00	.02	-.01	.03	-.02	.03
Location	.05	-.01	.03	.05	-.04	-.18***	.04	.05	-.03	-.01	.02
SES ladder	-.10***	-.12***	-.06	-.13***	-.14***	-.08*	-.30***	-.15***	-.13***	-.21***	-.11***
Education	.08**	.07*	.11***	.07*	.03	-.06	.04	-.00	.13***	.09**	.17***
Environ. social norms	.15***	.10***	.16***	.08**	.12***	.07*	.09**	.16***	.12***	.15***	.12***
***Adj R*** ^***2***^	.33	.18	.22	.29	.21	.08	.27	.24	.34	.29	.26
***F***	(9,980) 53.91***	(9,984) 25.53***	(9,990) 31.40***	(9,097) 46.81***	(9,990) 29.73***	(9,990) 10.44***	(9,990) 42.50***	(9,990) 36.24***	(9,986) 56.98***	(9,990) 46.77***	(9,990) 39.36***

N = 1000 per country; values are *β* coefficients; table of B coefficients, SEs, and CIs are available from the corresponding author.

* *p* < .05;

** *p* < .01;

*** *p* < .001

^a^. The appropriate within-country centered environmental attitudes and efficacy terms used to compute the respective interaction term.

EAV showed a significant association with PEBs in all countries except Kenya (*β* from .22 to .42). This one lack of significance corresponds with the finding that, in Kenya, the model only explained 8% of total variance in PEBs—less than a quarter to a half of that explained in any of the other countries. In comparison to the combined measure of EA tested on the overall sample (*β* from .16 to .55), EAV was less associated with PEBs (*β* from .05 to .42) in every country. EAV had the strongest association in the United Arab Emirates sample (*β* = .42, *p* < .001).

EFFS was significantly directly associated with PEBs in all of the countries except China. However, EFFS moderated the EAV-PEBPu relationship only in Brazil (*β* = -.11, *p* < .001).

## 6 Discussion

### 6.1 Summary

This study builds on prior research demonstrating relationships between EA, EFF, and PEB [e.g., 5–14, 17–19], by comparing models based on these constructs overall, with models based on the matching and mismatching subdimensions of these constructs, with efficacy taking a direct and moderating role, and across 11 countries. The results provide slightly stronger support for two models: (a) the simple model of EA, EFF, and PEBs (in particular, the direct effects version); and (b) a mismatch model with the subscales of EAV, EFFS, and PEBPu. Our expectations that subscales that were matched by domain (models 1 and 2) would explain more variance in PEBs than the mismatched models (3–8), were not supported. Instead, models that examined the effects of various subscales on PEBPu explained consistently more variance than models that used PEBPr. Similarly, models with EFFS as a predictor variable tended to explain more variance in PEBs than models with EFFC. Both EAC and EAV were good explanatory associations with PEBs, though the combined EA was noticeably stronger than either subscale alone. In particular, the specific mismatch model with EAV, EFFS, and PEBPu emerged as explaining the most variance in the overall sample, though only 2% more variance in PEBs than explained in the simple model (33% vs 31%).

Although the lower variance explained when testing PEBPr was not anticipated, there are two possible explanations for this pattern of results. First, with only two behavior items to capture engagement in PEBPr, these data do not represent the range of possible PEBPr that individuals in the sample may have considered or been able to perform. This problem is exacerbated by this study’s cross-country sample, in which diversity in contexts (such as recycling services) that influence participants’ engagement in various PEBs is expected [13, 39, 85, 86]. Second, because environmental problems cannot be solved by individual behaviors alone [71, 76, 81], it is possible that PEBPr are influenced by individuals’ EA and EFF to a lesser extent than are PEBPu, in which collective effort to achieve environmental goals is more salient. However, Tam and Chan [54] conducted a multi-level model of private and public PEBs across 32 countries and found that the partial correlation between environmental concern and PEBPu was *weaker* than that between environmental concern and PEBPr. Although their measures of PEBs were also limited, the contradictory results suggest that further research is needed to understand differential engagement PEBPu and PEBPr.

As expected, and supporting prior literature (e.g., [5–14]), EA were significantly associated with PEBs in both the overall analyses and across the majority of individual countries. Furthermore, when comparing the results of the simple model and the best-fit mismatch model by country (see Tables 4 and 5), it appears that the combined EA slightly outperformed each of the two EA subscales (EAC and EAV). The results suggest that when conducting cross-country research, a combined or comprehensive measure of pro-environmental attitudes may provide more explanation of individuals’ engagement in PEBs than measures of either domain alone. These findings support Kaiser et al.’s [26] argument that both environmental attitudes and behavior should be measured generally, because many specific influences and challenges to PEBs vary across individuals and contexts. Nevertheless, it is important to note that these findings do not completely contradict arguments for measuring behavior-specific attitudes, as discussed in the TPB and earlier theory of reasoned action [17, 21]. Still, in cross-country research, identifying PEB items and corresponding EAs at a granular level of specificity that are relevant to individuals in drastically diverse contexts may not be feasible or even useful, unless those specific behaviors are targets in environmental campaigns or interventions [3, 93].

There are several potential explanations for the weaker direct effects of EFF compared to EA. The first considers how efficacy was measured. The measure referred to global environmental issues and to long-term (10 years) outcomes. Individuals’ sense of efficacy in their ability to perform a small-scale, short-term pro-environmental task such as recycling in the coming week likely would have a stronger relationship with their engagement in that physically and temporally proximate behavior. Second, the role of some kinds of efficacy may be limited. In particular, the stronger relationships of EFFS compared to EFFC with PEBs suggest that the belief that one, personally, is capable of performing actions to achieve a goal is a stronger motivator of personal action than is the belief that one’s group can make a difference. These findings are consistent with previous research (see Section 2.2) that indicates that EFFC could eventually lead to inaction because a member may feel that their single behavior is unnecessary or insubstantial for goal achievement, relative to larger collection action [e.g., 94]. In addition, these results are consistent with findings that EFFS may be somewhat necessary for EFFC to influence individual behavior intentions, especially in large-scale environmental contexts [76], although the opposite relationship has also been reported [88].

The moderating role of EFF was not supported, either in the combined or in the mismatch models. As Table 3 shows, the simple moderation effect was non-significant (overall *β* = -.004) and the (mis)matched moderation effects ranged from a barely significant -.02 (*p* < .01), to a non-significant .01. Within countries (Table 4), the simple moderation effect was significant only in South Africa (*β* = -.08, *p* < .001), and in the mismatch model 5 only in Brazil (*β* = -.11, *p* < .001). Although a moderation role of efficacy has been found previously (e.g., [20]), our findings align with the larger body of research that shows no significant moderation [7, 11, 24, 45, 80]. Notably, these significant relationships were negative, which may suggest that a sense of efficacy to accomplish broader and more influential environmental goals may slightly undermine people’s drive to engage in the smaller-scale PEBs measured in this study.

When considered separately by country, the results indicate that countries share much in common, but that there are still some diverse strengths and even diverse directions of relationships between these variables. EA and EFFS were consistently related to PEBs, with one exception each in two countries. These findings align with a few previous international studies that demonstrate consistent influences of EA, EFF, and norms on PEBs across some countries (Section 3.4) (e.g., [9, 29,30]). Furthermore, descriptive environmental social norms and the SES ladder maintained nearly consistent relationships with PEBs across countries; however, as a regression block, demographics explained a wide range of unique variance (3.8% to 11.6%) in PEBs across countries. This variation likely reflects some of the different contextual factors in each country, as generally noted in Section 3.4 (e.g., [12, 13, 85, 86]).

The consistency of relationships between these model variables across the countries suggests that theories applying these variables, including rational choice models such as the TPB, and pro-social behavioral models such as the norm activation model and value-belief-norm model [25], are likely to be applicable (if somewhat unevenly) across countries [6].

Results of the simple and (mis)match models demonstrate that country-level differences (admittedly aggregated and unidentified) are not as influential on PEBs as are EA, and, to a slight extent, EFF. In the simple model, country-level influences explained only 6% of unique variance in PEBs, though in the (mis)match models, country differences explained a consistently greater 12%-14% of PEB variance. It appears that specifying the domain (specific or general) of PEBs allows country-level effects to emerge in the model to a greater extent than does using the combined measure of PEBs. Although prior cross-country studies have demonstrated consistency in the relationships between EA, PEB, and EFF across countries (e.g., [9, 29, 30,]), this study is the first that we are aware to suggest that country-level effects are more easily detected when examining the subdimensions of these constructs.

Concerning demographics, age, location, and education had significant associations with PEBs in only some countries. Age had a positive significant association on in Brazil, South Africa, South Korea, and Indonesia. Location mattered only in five countries, and negatively so only in Kenya, where respondents in more rural areas engaged in more public PEBs (*β* = -.15, *p* < .001). Education had a positive significant influence in six countries, but, unexpectedly, a negative association (*β* = -.07, *p* < .001), also in Kenya. The SES ladder had a significant negative association in all but one country (again, Kenya), while descriptive environmental social norms had a significant positive influence in all countries. Therefore, it is clear that these additional contextual variables should be taken into account when examining or attempting to motivate PEB locally. Policies or communication efforts concerning EA, EFF, or PEBs in different countries should be tailored accordingly.

### 6.2 Limitations and future directions

These findings are subject to several limitations. As noted in Section 2.2, there is a wide range of other direct, moderating, and mediating individual-level covariates associated with PEBs [29, 95, 96]. Gifford [3], for example, briefly reviews a wide range of influences and constraints on PEB, such as “childhood experience; knowledge and education; personality; perceived behavioral control; values, attitudes, and worldviews of various kinds; felt responsibility and moral commitment; place attachment; norms and habits; goals; affect; and many demographic factors” (p. 544). PEBs can be affected by routine, constraints, perceived small effect, awareness of effects, non-environmental considerations, etc.; more generally, by aspects of actor, context, and behavior [25], political ideology [97], and religious affiliation [98]. Other contextual forces include interpersonal, community expectations, media, regulation, institutional policies, incentives and costs, difficulty, built environment, public policies, and social, economic and political aspects, all of which may be differentially salient or interpreted [25]. Therefore, the strength of the relationships among measured variables are likely to vary when additional covariates are considered.

As noted, the literature is somewhat vague about distinctions among specific and general subdimensions of attitudes, efficacy, and pro-environmental behaviors. We have explicated these distinctions, based on prior theory and research, for the purposes of the mismatching/matching analyses. However, both the conceptual distinctions, and, as noted, the particular items used to measure these, may be improved and further tested in future research.

Furthermore, as mentioned above, the nature of this secondary analysis involves typical strengths and weaknesses of measures and data from a prior project. Copious research on environmental issues has led to a wide variety of measures of pro-environmental attitudes, in some cases designed for specific contexts or behaviors. Gifford and Sussman [29], noting at least 15 measures, highlight the 120-item, 12-subscale, Environmental Attitudes Inventory (EAI) [28]. Thus, the use of more extensive and validated measures of environmental attitude such as the EAI or the New Environmental Paradigm (NEP) [99, 100] could strengthen confidence in these findings. Certainly future research could consider other manifestations of environmental values, such as postmaterialism, altruism, or free market processes [25, 29] or instrumental, intrinsic, and relational facets [89, 101]. Additional environmental values measures emphasize different conceptualizations [28, 102, 103].

Additionally, PEBs were measured with only five items, a list that is both too short and not equally applicable for every participant. Broader measures of PEBs that capture a range of relevant cross-country public and private behaviors [6, p. 290, 104–106] can clarify differential engagement in the same PEBs across countries. Furthermore, self-reported PEBs do not necessarily correspond highly to actual PEBs, with an average association of r = .45 [3]; however, our measures did ask for PEBs in the past 12 months instead of intended PEBs, which is a strength of this study. The items used to measure the EFF scale and subscales also have incomplete construct validity; future studies should consider employing the validated scale developed by Moeller and Stahlmann [107] or measuring efficacy in terms of the specific PEBs items used in this study.

Finally, with a sample of only 11 countries, and the focus of this study on the consistency of the models overall and across countries, rather than on hypothesizing, explaining, or testing for country differences, we can only speculate as to the (apparently somewhat limited) country-level factors that influence these relationships. Scholars have examined many country-level influences, such as postmaterialist values [12], human development indices [106, 108], cultural and psychological distance [109], and individualism vs. collectivism or other cultural values [54, 106, 110, 111], all of which indicate that country-level contexts can facilitate or hinder PEBs. Future research on PEBs and their antecedents across a sufficient number of countries for multi-level modeling, applying relevant country-level measures [e.g., 30, 54], can provide insight into the country-level contexts that affect the relationships between EA, EFF, and PEBs. As practitioners seek to promote increased engagement in PEB, interventions and promotions should be based on these detailed nuances of determinants of pro-environmental behavior, matching interventions to determinants [112] and taking into account implications from meta-analyses [113].

## 7 Conclusion

Understanding how the relationships between environmental attitudes, efficacy, and pro-environmental behaviors vary (or are similar) around the world adds important nuance to our theoretical and practical knowledge base. Counter to expectations based on prior research, we find that models that match pro-environmental attitude, environmental efficacy, and pro-environmental behavior variables on domain (specific or general) do not necessarily outperform simple models or some mismatch models. Further, while the relationships are fairly consistent across countries, variations do exist and may be useful in helping to shape environmental communication strategies in different countries. Together, these findings point to pro-environmental attitudes as being the strongest link to pro-environmental behaviors, both overall and within diverse countries.

More and better promotion of local and global engagement in pro-environmental behaviors is urgently needed, in accord with continuous reporting about the degradation of the environment and increasing consequences of climate change. Results from this study suggest that interventions should center on strengthening these core relationships while simultaneously accounting for local contextual factors. Additional research that examines sufficient countries for multi-level modeling can further shed light on the contextual constructs that should be considered when developing pro-environmental interventions.

## Supporting information

S1 File (DOCX)
