# Supplementary material for: (Mis)matched direct and moderating relationships among pro-environmental attitudes, environmental efficacy, and pro-environmental behaviors across and within 11 countries
Source: PLoS One. 2024 Jun 18;19(6):e0304945. doi: 10.1371/journal.pone.0304945 (PMC11185491; doi:10.1371/journal.pone.0304945)
Supplement: S1 File — (DOCX) [file pone.0304945.s001.docx]

**S0_File**: Supporting Information: Methodological Notes for (Mis)matched direct and moderating relationships among pro-environmental attitudes, environmental efficacy, and pro-environmental behaviors across and within 11 countries.

**S1. Efficacy Review: Self/Collective.**

**S2. The Absence of Mediation Analyses.**

**S3. Subscale and Scale Dimensionality (EFA and CFA), Reliability, Discriminant Validity, and Cross-Country Factor Congruence of Measures.**

**S3.1. Exploratory Factor Analysis Loadings of Separate Subscales.**

**S3.1a. Table. Exploratory Factor Analysis Loadings of Separate Subscales.**

**S3.1b. Table. Exploratory Factor Analysis Loadings of All Subscale Items.**

**S3.2. Confirmatory Factor Analysis Loadings and Fit for Subscales and Scales.**

**S3.2 Fig. CFA Models for Subscales and Scales.**

**S3.2 Table. Confirmatory Factor Analysis Standardized Factor Loadings and Fit Measures for Subscales and Scales (using Amos v. 24).**

**S3.3. Discriminant Validity.**

**S3.3a. Table. Discriminant Validity among Scales and Subscales, Overall: AVE Approach. S3.3b. Table. Discriminant Validity among Subscales, Overall: HTMT Approach.**

**S3.4. Reliabilities.**

**S3.4 Table. Subscale and Scale Reliabilities (FAr and Cronbach).**

**S3.5. Scale Factor Loadings and Congruence across Countries.**

**S3.5 Table. Exploratory Factor Loadings and Factor Congruence for Combined Measures, Overall and by Country.**

**S3.6. Summary.**

**S4. Taking into Account Country-level Effects in the Overall Analyses.**

**S4.1 Table. Tests for Overall Mean Differences, Robust Errors Mean Test, Homogeneity of Variance, and Intra-Cluster Coefficients across 11 Countries.**

**S5. Statistical Differences across Models and Countries.**

**S6. Definitions and Abbreviations of Terms Used in Text.**

**S6.1. Table. Definitions and Abbreviations of Terms Used in Text.**

**S7. References.**

**S1. Efficacy Review: Self/Collective**

Self- and collective efficacy represent different, though related, concepts. Collective efficacy is theoretically distinct from, and can influence, self-efficacy [1, studying teachers' efficacy]. Some studies consider only collective efficacy. For example, Landmann and Rohmann [2] distinguished collective efficacy as people’s appraisals of their perceptions of a behavior: “People can be positively moved by the belief that they can achieve something together, and this can motivate their intention to act collectively in the future” (p. 9). They found a positive relation between collective efficacy and collective action related to forest protection (esp. those actions requiring less effort), mediated by the emotion of “being moved.” Other studies show independent effects of the two kinds of efficacy. Fernández‐Ballesteros et al. [3] differentiated between personal/individual and collective efficacy, finding that the former was positively related to perceived ability to manage worklife, relationships, and financial condition, and the latter associated with achieving social change through joint action. Jugert et al. [4] reviewed literature discriminating self-efficacy from collective efficacy, and empirically showed that both can positively influence pro-environmental behavior intentions. Some studies report relationships between self- and collective efficacy. For instance, in an educational setting, Versland and Erickson [5] showed that both a school’s principal’s self-efficacy (leadership, focus) and the school’s collective efficacy (e.g., initiative, staff relations) were associated with teachers’ self-efficacy. Roos et al.’s [6] analysis of surveys from Setswana, South African respondents indicated that collective efficacy and self-efficacy were positively associated with each other and with psychological well-being. In student engineering project teams, collective efficacy was associated with team cohesion, team performance, and personal self-efficacy, and had a stronger relationship with team performance than did self-efficacy [7].

**S2. The Absence of Mediation Analyses**

As noted in the text, we did not pursue the role of efficacy as a mediator. Most theoretical justification for the mediational role of environmental efficacy comes from other areas besides environmental psychology or communication, such as health behavior (e.g., [8]). These scholars point to the extended parallel process model, the health belief model, PMT, and SCT to demonstrate mediation pathways (see brief review by Knerr et al. [9]). A few significant mediation results have been found in the environmental literature (e.g., [10–15]). For example, Anker et al. [16] did not find that self-efficacy (one of three components of “vested interest”) moderated the relationship between positive attitudes and prosocial donations, but did show that it mediated the relationship for organ/tissue and blood donation. Jugert et al. [4] reviewed research showing that collective efficacy interventions can positively influence pro-environmental behavior intentions, through both collective and self-efficacy. Hurst Loo and Walker [17] showed that efficacy mediated the relationships between pro-environmental knowledge and attitude toward climate change mitigation. Because of the lack of much research on efficacy as a mediator in environmental research, the associated expansion in the number of analyses that would be required, the cross-sectional nature of this study’s data, and critiques of mediation analyses [18,19], we do not pursue the mediation approach here.

**S3. Subscale and Scale Dimensionality (EFA and CFA), Reliability, Discriminant Validity, and Cross-Country Factor Congruence of Measures**

Although, as the text notes, the measures were not designed by us, and do not reflect standard measures in the literature, we still need to verify that the scales can be operationalized as unidimensional, reliable, and congruent across countries. Thus, we report results and justifications from Exploratory Factor Loadings of Separate Subscales, Exploratory Factor Loadings of all Subscale Items, Confirmatory Factor Analysis Loadings and Fit for Subscales and Scales, Two Forms of Scale Reliabilities, Two Forms of Discriminant Validity, Scale Factor Loadings and Congruence across Countries, and Taking into Account Country-level Effects, and end with a Summary.

**S3.1 Exploratory Factor Analysis Loadings of Separate Subscales**

Table S3.1a shows that each separate subscale is unidimensional, consisting of sufficiently high loadings (from .745 to .889).

**Table S3.1a. Exploratory Factor Analysis Loadings of Separate Subscales.**

| Environmental Concern (EAC) |  |
| --- | --- |
| Q13_1 Habitat loss | .785 |
| Q13_2 Plastic pollution | .795 |
| Q13_3 Global climate change | .791 |
| Q13_4 Species at risk of extinction | .773 |
| Q13_5 Air pollution | .823 |
| Q13_7 Lack of clean drinking water | .745 |
| *Eigenvalue* | 3.70 |
| *% Variance (AVE)* | 61.7 |
| *Kaiser-Meyer-Olkin measure of sampling adequacy* | .886 |
| *Bartlett’s test of sphericity* | *p*<.000 |
| *Joreskog rho reliability* | .91 |
| Environmental Values (EAV) |  |
| Q3_2 Conserving natural resources is important for the country’s economy | .786 |
| Q3_3 Conserving nature is a reflection of my core moral beliefs and convictions | .766 |
| Q3_4 Nature is important to me, to who I am as a person | .804 |
| Q3_5 Protecting nature is important for people’s health | .804 |
| Q3_6 Being in/seeing nature brings people pleasure or satisfaction | .771 |
| *Eigenvalue* | 3.09 |
| *% Variance (AVE)* | 61.8 |
| *Kaiser-Meyer-Olkin measure of sampling adequacy* | .857 |
| *Bartlett’s test of sphericity* | *p*<.000 |
| *Joreskog rho reliability* | .89 |
| Self-Efficacy (EFFS) |  |
| Q14_1 Protect habitats | .875 |
| Q14_2 Reduce plastic pollution in our oceans | .802 |
| Q14_3 Reduce use of fossil fuels (e.g., petroleum, natural gas, coal) | .804 |
| Q14_4 Save animals at risk of extinction | .849 |
| *Eigenvalue* | 2.78 |
| *% Variance (AVE)* | 69.4 |
| *Kaiser-Meyer-Olkin measure of sampling adequacy* | ,783 |
| *Bartlett’s test of sphericity* | *p*<.000 |
| *Joreskog rho reliability* | .90 |
| Collective Efficacy (EFFC) |  |
| Q15_1 Protect habitats | .889 |
| Q15_2 Reduce plastic pollution in our oceans | .859 |
| Q15_3 Reduce use of fossil fuels (e.g., petroleum, natural gas, coal) | .830 |
| Q15_4 Save animals at risk of extinction | .876 |
| *Eigenvalue* | 2.98 |
| *% Variance (AVE)* | 74.6 |
| *Kaiser-Meyer-Olkin measure of sampling adequacy* | .820 |
| *Bartlett’s test of sphericity* | *p*<.000 |
| *Joreskog rho reliability* | .92 |
| Private PEBs (PEBPr) |  |
| Q17_1 Recycle | .845 |
| Q17_3 Use your own reusable shopping bags | .845 |
| *Eigenvalue* | 1.43 |
| *% Variance (AVE)* | 71.5 |
| *Kaiser-Meyer-Olkin measure of sampling adequacy* | .50 |
| *Bartlett’s test of sphericity* | *p*<.000 |
| *Joreskog rho reliability* | .83 |
| Public PEBs (PEBPu) |  |
| Q17_2 Avoid products with ingredients that are bad for the environment | .790 |
| Q17_5 Talk to friends or family about an environmental issue | .856 |
| Q17_6 Used social media to share information about an environmental issue | .820 |
| *Eigenvalue* | 2.03 |
| *% Variance (AVE)* | 67.7 |
| *Kaiser-Meyer-Olkin measure of sampling adequacy* | .682 |
| *Bartlett’s test of sphericity* | *p*<.000 |
| *Joreskog rho reliability* | .86 |
| N=10,999  Note: Principal Component Analyses; each separate extraction provided one component. | |

Table 3.1b confirms that all items load on their separate subscales when analyzed together. This also provides support for discriminant validity (see S3.3 below).

**Table 3.1b. Exploratory Factor Loadings of All Subscale Items.**

|  | **Component/Construct** | | | | | |
| --- | --- | --- | --- | --- | --- | --- |
| **Items** | **EAC** | **EAV** | **EFFC** | **EFFS** | **PEBPu** | **PEBPr** |
| Q13_1 HabitatLoss | **.731** | .212 | -.008 | .070 | .104 | .131 |
| Q13_2 PlasticPollution | **.753** | .229 | .023 | .042 | .033 | .139 |
| Q13_3 GlobalClimateChange | **.757** | .190 | .029 | .080 | .135 | .018 |
| Q13_4 SpeciesAtRiskOfExtinction | **.712** | .214 | .006 | .086 | .135 | .117 |
| Q13_5 AirPollution | **.787** | .234 | .029 | .065 | .072 | .016 |
| Q13_7 LackOfCleanDrinkingWater | **.730** | .188 | .027 | .036 | .090 | -.071 |
| Q3_2 Conserving natural resources is important for the country’s economy | .277 | **.732** | .039 | .056 | .058 | .049 |
| Q3_3 Conserving nature is a reflection of my core moral beliefs and convictions | .216 | **.695** | .064 | .047 | .243 | .046 |
| Q3_4 Nature is important to me, to who I am as a person | .236 | **.733** | .030 | .103 | .228 | .020 |
| Q3_5 Protecting nature is important for people’s health | .297 | **.743** | .029 | .070 | .050 | .045 |
| Q3_6 Being in/seeing nature brings people pleasure or satisfaction | .228 | **.745** | .030 | .052 | -.003 | .127 |
| Q14_1 SelfE Protect habitats | .064 | .095 | .260 | **.815** | .163 | -.041 |
| Q14_2 SelfE Reduce plastic pollution in our oceans | .131 | .102 | .255 | **.762** | -.046 | .124 |
| Q14_3 SelfE Reduce use of fossil fuels (e.g., petroleum, natural gas, coal) | .105 | .054 | .258 | **.744** | .085 | .089 |
| Q14_4 SelfE Save animals at risk of extinction | .039 | .057 | .279 | **.770** | .244 | -.066 |
| Q15_1 CollE Protect habitats | .016 | .068 | **.844** | .259 | .043 | -.008 |
| Q15_2 CollE Reduce plastic pollution in our oceans | .023 | .040 | **.827** | .232 | -.017 | .069 |
| Q15_3 CollE Reduce use of fossil fuels (e.g., petroleum, natural gas, coal) | .017 | -.005 | **.811** | .197 | .039 | .074 |
| Q15_4 CollE Save animals at risk of extinction | .019 | .063 | **.830** | .260 | .084 | -.056 |
| Q17_1 Recycle | .090 | .068 | .010 | .058 | .110 | **.820** |
| Q17_3 Use your own reusable shopping bags | .092 | .105 | .049 | .006 | .130 | **.792** |
| Q17_2 Avoid products with ingredients that are bad for the environment | .188 | .200 | .051 | .120 | **.641** | .323 |
| Q17_5 Talk to friends or family about an environmental issue | .183 | .185 | .041 | .126 | **.771** | .178 |
| Q17_6 Used social media to share information about an environmental issue | .117 | .092 | .047 | .127 | **.843** | -.038 |

N=10,999

Note: Extraction Method: Principal Component Analysis; Rotation Method: Varimax with Kaiser Normalization; Rotation converged in 6 iterations; bold values are loadings corresponding to the constructs in the column headings; Kaiser-Meyer-Olkin measure of sampling adequacy = .896; Bartlett’s test of sphericity *p*<.000

**S3.2 Confirmatory Factor Analysis Loadings and Fit for Subscales and Scales**

We conducted confirmatory factor analysis for each subscale and scale, using Amos v. 24 (with ML estimation). Hu and Bentler [20] recommend reporting combinations of measures; Kline [21] argues for reporting χ2 , CFI, and SRMR. Gaskin and Lim [22] prefer a combination of CFI>0.95 and SRMR<0.08, with additional evidence provided by RMSEA<0.06. Table S3.2 provides the standardized loadings and the fit measures for each analysis. Because of the very large sample size, all measures involving χ2 will typically be large and highly significant.

Note that for the combined scales (EA, EFF, PEBs) we are not testing for a second-order factor. We consider that the full scales can each be treated as unidimensional, each consisting of two unidimensional subscales. Thus, in the CFA analyses of the simple models, we link the two subscales, each with their own item indicators, via covariance between the two subscales. Fig S3.2 presents the visual model for each CFA.

**Fig S3.2. CFA Models for Subscales and Scales**

With some modifications, the models exhibited the following fit measures: for AGFI > .9, achieved for all analyses; for CFI > .95, achieved for all analyses; for RMSEA < .06, achieved for 4 of 7 analyses; and for SRMR < .08, achieved for all analyses.

**Table S3.2. Confirmatory Factor Analysis Standardized Factor Loadings and Fit Measures for Subscales and Scales (using Amos v. 24).**

| Item | EAV | EAC | EA | EFFS | EFFC | EFF | PEBPr | PEBPu | PEBs |
| --- | --- | --- | --- | --- | --- | --- | --- | --- | --- |
| q3_2 | .723 |  | .727 |  |  |  |  |  |  |
| q3_3 | .694 |  | .691 |  |  |  |  |  |  |
| q3_4 | .748 |  | .743 |  |  |  |  |  |  |
| q3_5 | .750 |  | .756 |  |  |  |  |  |  |
| q3_6 | .702 |  | .699 |  |  |  |  |  |  |
| q13_1 |  | .684 | .692 |  |  |  |  |  |  |
| q13_2 |  | .752 | .753 |  |  |  |  |  |  |
| q13_3 |  | .755 | .751 |  |  |  |  |  |  |
| q13_4 |  | .669 | .679 |  |  |  |  |  |  |
| q13_5 |  | .809 | .804 |  |  |  |  |  |  |
| q13_7 |  | .694 | .692 |  |  |  |  |  |  |
| q14_1 |  |  |  | .894 |  | .881 |  |  |  |
| q14_2 |  |  |  | .647 |  | .658 |  |  |  |
| q14_3 |  |  |  | .644 |  | .657 |  |  |  |
| q14_4 |  |  |  | .830 |  | .836 |  |  |  |
| q15_1 |  |  |  |  | .887 | .885 |  |  |  |
| q15_2 |  |  |  |  | .756 | .754 |  |  |  |
| q15_3 |  |  |  |  | .707 | .704 |  |  |  |
| q15_4 |  |  |  |  | .858 | .863 |  |  |  |
| q17_1 |  |  |  |  |  |  | -- |  | .663 |
| q17_3 |  |  |  |  |  |  | -- |  | .652 |
| q17_2 |  |  |  |  |  |  |  | -- | .663 |
| q17_5 |  |  |  |  |  |  |  | -- | .815 |
| q17_6 |  |  |  |  |  |  |  | -- | .680 |
| Fit | EAV | EAC | EA | EFFS | EFFC | EFF | PEBPr | PEBPu | PEBs |
| χ2 (df) | 311.8 *** (5) | 274.2 ***  (8) | 835.3  ***  (42) | 23.4 ***  (1) | .43  ns  (1) | 2029.3 ***  (16) | too few items | too few items | 639.98 ***  (4) |
| χ2 /df | 62.4 *** | 34.28 *** | 19.8  *** | 23.4 *** | .43 | 126.8  *** | -- | -- | 159.99 *** |
| AGFI | .966 | .979 | not computed | .989 | 1.00 | .905 | -- | -- | .916 |
| CFI | .985 | .991 | .986 | .999 | 1.00 | .962 | -- | -- | .949 |
| RMSEA | .075 | .055 | .041 | .045 | .000 | .107 | -- | -- | .120 |
| SRMR | .0207 | .0168 | .0201 | .0049 | .0006 | .0356 | -- | -- | .0489 |
| Latent variable corrs |  |  | EAC-EAV:  .27 |  |  | EFFS-EFFC:  .63 |  |  | PEBPr- PEBPu: .48 |
| Modifications with error covariances | -- | e13.1-e13.4: .32 | e13.1-e13.4:  .30 | e14.2-e14.3:  .28 | e15.2-e15.3:  .25 | e14.2-e14.3: .27  e15.2-e15.3: .25  e14.3 – e15.7: .34 | -- | -- | -- |

N=11,000; *** p < .001

Note: See Table S3.1 for item wordings.

**S3.3 Discriminant Validity**

Table S3.3a provides results of the standard approach to verifying discriminant validity [23]. Bolded values on the diagonal are AVE (average variance extracted); they are all higher than the squared correlations between their respective specific constructs, and much higher than the squared correlations with the other combined, specific, and general constructs, indicating discriminant validity. The AVEs of the specific measures are less than the squared correlations with the combined constructs, indicating that the specific or general versions are subsets of the combined constructs.

**Table S3.3a. Discriminant Validity among Subscales, Overall: AVE Approach**

| **Construct** | **EAC** | **EAV** | **EFFS** | **EFFC** | **PEBPr** | **PEBPu** |
| --- | --- | --- | --- | --- | --- | --- |
| EAC | **.617** |  |  |  |  |  |
| EAV | .336 | **.618** |  |  |  |  |
| EFFS | .048 | .058 | **.694** |  |  |  |
| EFFC | .006 | .017 | .325 | **.746** |  |  |
| PEBPr | .048 | .053 | .014 | .006 | **.715** |  |
| PEBPu | .137 | .160 | .102 | .023 | .102 | **.677** |

Note: N=11,000; Diagonal values are Average Variances Explained (AVE); off-diagonal values are squared Pearson correlations (see Table 2 in the text); values in boxes are from specific/general subscales of the respective combined construct.

Table S3.3b provides the results from a recent alternative approach, the heterotrait-monotrait ratio of correlations (HTMT) [24].

**Table S3.3b. Discriminant Validity among Subscales, Overall: HTMT Approach**

| **Construct** | **EAC** | **EAV** | **EFFS** | **EFFC** | **PERPr** | **PEBPu** |
| --- | --- | --- | --- | --- | --- | --- |
| EAC | -- |  |  |  |  |  |
| EAV | **.6773** | -- |  |  |  |  |
| EFFS | .2564 | .2747 | -- |  |  |  |
| EFFC | .0956 | .1488 | **.6556** | -- |  |  |
| PERPr | .3082 | .3187 | .1628 | .1061 | -- |  |
| PEBPu | .4574 | .5020 | .4027 | .1872 | **.4907** | -- |

Note: N=11,000; Bolded values are HTMT values; values in boxes indicate extent of construct discriminant validity from specific/general subscales of the respective combined construct; other values are between subscales from different combined constructs; satisfactory values are below .85.

**S3.4 Reliabilities**

After determining unidimensionality, we turn to scale and subscale reliability. The traditional measure from EFA is Cronbach’s α. Hayes and Coutts [25] propose using a more general reliability measure “omega” (*ω*) instead of α for estimating reliability, show how it is computed in SEM and R programs, and provide a macro for SPSS or SAS which does not require CFA loadings or error variances but relies on maximum likelihood principal component analysis (EFA-ML). Their analyses show, though, that both α and *ω* produce nearly identical values. However, Cho [26] does not like *ω* (nor α), stating that “There is little *empirical* evidence that FA reliability is more accurate than non-FA reliability.... currently available empirical findings offer evidence against the accuracy and conservatism of FA reliability....”, and that there is little assessment of how small sample sizes or sampling errors affect FA type reliability estimators. Finally, Cho points out that *ω* really refers to a variety of FA reliability estimators, so it is inappropriate to refer to this measure as omega; rather, it should be referred to as *FAr* (factor analysis reliability).

Table S3.4 shows, indeed, that the *FAr* (omega or *ω*) and Cronbach’s α for all scales and subscales are almost exactly the same.

**Table S3.4. Subscale and Scale Reliabilities (FAr and Cronbach).**

| **Subscales and Scales** | **# Items** | **EFA-ML *FAr*** | **Cronbach α** |
| --- | --- | --- | --- |
| Environmental concern (EAC) | 6 | .875 | .875 |
| Environmental values (EAV) | 5 | .845 | .844 |
| Combined Pro-environmental attitude (EA) | 11 | .896 | .895 |
| Self-efficacy (EFFS) | 4 | .854 | .853 |
| Collective efficacy (EFFC) | 4 | .887 | .886 |
| Combined efficacy (EFF) | 8 | .885 | .889 |
| Private PEBs (PEBPr) | 2 | Too few items | .601 |
| Public PEBs (PEBPu) | 3 | .762 | .755 |
| Combined PEBs (PEBs) | 5 | .723 | .712 |

Note: Using SPSS Macro for omega reliability (Hayes & Coutts, 2020, 2022).

**A3.5 Scale Factor Loadings and Congruence across Countries**

Now that we have established appropriate dimensionality and reliability of the subscales and scales, we can consider whether the factor loadings are consistent across countries.

Lorenzo-Seva and Berge [27] note that while multigroup CFA is commonly used to test equivalence of factors, “when the sample size is large, any hypothesis of equal factors will systematically be rejected. Moreover, the available software for CFA often fails to converge to a solution” (p. 57) (though that was in 2006). It is also difficult to test complex factor structures. Finally, for the current study, there are six subscales for the overall sample and each of the 11 countries; across all invariance tests that would involve a very large number of group comparisons. Thus, we choose to test only congruence of the single overall factor for each of the three main constructs, for each country compared to the overall sample.

Lorenzo-Seva and Berge [27] propose the Tucker’s congruence coefficient (often referred to as Phi or ϕ), and provide an empirical basis for categorizing values as terrible, poor, borderline, good, or excellent. Milfont et al. [28] used Tucker’s Phi to support a uni-dimensional factor invariance of the value of social dominance orientation across countries. Fortunately, DeCoster [29] provided a spreadsheet to use in computing this measure. They also note that prior Procrustes rotations of the factors maximizes the congruence coefficient, but that approach does not seem to be used much. For example, the Błachnio et al. [30] article does not, nor does the DeCoster [29] spreadsheet, but the Milfont et al. [28] does.

Thus one could use OrthoSim (<https://www.pbarrett.net/orthosim/orthosim.html>) [31], which offers orthogonal Procrustes rotation before then computing four congruence measures. Barrett [32] shows in detail how neither a Pearson correlation or the Tucker coefficient is fundamentally valid, concluding that “I will no longer use the congruence coefficient any more unless its value is corroborated by using an index which is sensitive to loading/coordinate magnitudes as well as monotonic relations, i.e., Double-scaled Euclidean [DESD] or the kernel smoothed distance [KSD] measures of agreement. However, it is probably fair to say that most factor solutions which have previously used the congruence coefficient are probably Ok - in that the loadings are invariably of similar size and scale and so its value could be considered a fair representation of the agreement between two vectors” (p. 12); “I recommend only using a congruence coefficient as a matching index alongside a DSED or KSD coefficient” (p. 55), and “when using raw factor loading comparisons (where no procrustean row normalization has been requested), never use a congruence coefficient now without confirming the validity of its value using a distance function alongside it” (p. 74). However, there are no levels or rankings associated with the two distance/similarity measures, so it is difficult to actually apply those as criteria.

Lovik et al. [33] focus on that sensitivity to difference in signs of comparison loadings (which Lorenzo-Seva & Berge [27] also noted), overestimation when signs of the loadings across the factor pairs are mostly the same, underestimation if signs are mostly different, problems when using different samples because the order of the factors may be different and thus not consistent with the comparison matrix (vector), and problems if items are negatively framed (incorrect if not reversed, likely low even if reversed). They attempt to resolve some of these problems by offering a modified Tucker’s congruence coefficient. Referred to as mϕ (modified ϕ*)*, this uses the absolute value of the products in the numerator. Thus, there is no direct relationship between phi and mϕ, but is similarly only valid for a simple factor structure. However, when cross-loadings are high, near or above primary loadings, or primary loadings are low, neither coefficient works well (though the modified congruence values are slightly higher), though this is typically not the situation for a single factor structure. For the modified measure they propose .95 as the acceptable threshold. They also conclude that “it is quite problematic to set cut-off values that are valid in every situation,” so both the standard and the modified Tucker’s coefficients “should be interpreted and used with caution.”

Table S3.5 shows, indeed, that the factor loadings for each country are extremely congruent with the overall factor loadings. As all loadings are high and positive, using mϕ is unnecessary.

**Table S3.5. Exploratory Factor Loadings and Factor Congruence for Combined Measures, Overall and by Country.**

|  | **ALL** | **US** | **Mex** | **Bra** | **UK** | **SA** | **Ken** | **Chi** | **SK** | **Aus** | **UAE** | **Indo** |
| --- | --- | --- | --- | --- | --- | --- | --- | --- | --- | --- | --- | --- |
| **EA** |  |  |  |  |  |  |  |  |  |  |  |  |
| Q3 2 | .683 | .689 | .638 | .668 | .713 | .644 | .570 | .655 | .693 | .704 | .676 | .619 |
| Q3 3 | .652 | .718 | .631 | .373 | .722 | .708 | .614 | .635 | .660 | .737 | .668 | .574 |
| Q3 4 | .688 | .691 | .667 | .676 | .724 | .710 | .584 | .663 | .541 | .750 | .687 | .588 |
| Q3 5 | .705 | .738 | .663 | .698 | .740 | .649 | .541 | .652 | .721 | .754 | .710 | .636 |
| Q3 6 | .651 | .638 | .624 | .637 | .702 | .624 | .583 | .659 | .682 | .685 | .671 | .608 |
| Q13 1 | .720 | .780 | .736 | .728 | .775 | .723 | .645 | .657 | .694 | .789 | .655 | .771 |
| Q13 2 | .732 | .736 | .730 | .737 | .780 | .710 | .659 | .702 | .734 | .776 | .733 | .721 |
| Q13 3 | .720 | .719 | .723 | .746 | .696 | .699 | .721 | .722 | .770 | .630 | .709 | .731 |
| Q13 4 | .715 | .750 | .730 | .783 | .729 | .708 | .634 | .624 | .702 | .773 | .714 | .696 |
| Q13 5 | .756 | .788 | .745 | .779 | .728 | .730 | .688 | .733 | .775 | .715 | .772 | .772 |
| Q13 7 | .675 | .662 | .719 | .755 | .593 | .659 | .550 | .579 | .705 | .609 | .713 | .695 |
| *Eig* | 5.40 | 5.71 | 5.28 | 5.35 | 5.70 | 5.21 | 4.22 | 4.84 | 5.40 | 5.74 | 5.41 | 5.05 |
| *% Var* | 49.1 | 51.9 | 48.0 | 48.7 | 51.8 | 47.4 | 38.4 | 44.0 | 49.1 | 52.2 | 49.2 | 45.9 |
| *Congr* | -- | .999 | .999 | .991 | .998 | .999 | .998 | .999 | .997 | .997 | .999 | .998 |
| **EFF** |  |  |  |  |  |  |  |  |  |  |  |  |
| Q14 1 | .758 | .744 | .755 | .739 | .729 | .720 | .720 | .818 | .747 | .750 | .749 | .799 |
| Q14 2 | .705 | .731 | .691 | .645 | .695 | .622 | .633 | .793 | .674 | .709 | .795 | .738 |
| Q14 3 | .703 | .718 | .664 | .666 | .683 | .635 | .680 | .754 | .678 | .736 | .717 | .745 |
| Q14 4 | .746 | .717 | .721 | .725 | .705 | .713 | .704 | .804 | .734 | .732 | .763 | .814 |
| Q15 1 | .803 | .822 | .782 | .841 | .792 | .790 | .711 | .839 | .801 | .816 | .789 | .838 |
| Q15 2 | .766 | .787 | .775 | .803 | .787 | .791 | .593 | .831 | .719 | .800 | .766 | .792 |
| Q15 3 | .733 | .768 | .683 | .774 | .757 | .763 | .630 | .776 | .706 | .775 | .725 | .770 |
| Q15 4 | .797 | .832 | .781 | .827 | .811 | .768 | .673 | .850 | .778 | .806 | .770 | .828 |
| *Eig* | 4.53 | 4.70 | 4.30 | 4.57 | 4.46 | 4.24 | 3.59 | 5.20 | 4.28 | 4.70 | 4.62 | 5.01 |
| *% Var* | 56.6 | 58.7 | 53.7 | 57.1 | 55.7 | 53.0 | 44.8 | 65.4 | 53.4 | 58.8 | 57.7 | 62.6 |
| *Congr* | -- | .999 | .999 | .999 | .999 | .999 | .998 | .999 | .999 | .999 | .999 | .999 |
| **PEB** |  |  |  |  |  |  |  |  |  |  |  |  |
| Q17 1 | .565 | .580 | .747 | .725 | .384 | .747 | .640 | .678 | .610 | .443 | .718 | .703 |
| Q17 2 | .784 | .824 | .743 | .765 | .827 | .794 | .655 | .742 | .744 | .813 | .755 | .757 |
| Q17 3 | .568 | .694 | .722 | .713 | .245 | .685 | .328 | .676 | .687 | .310 | .725 | .690 |
| Q17 5 | .797 | .807 | .770 | .791 | .824 | .774 | .799 | .703 | .778 | .830 | .784 | .774 |
| Q17 6 | .695 | .656 | .683 | .715 | .671 | .707 | .709 | .666 | .666 | .693 | .736 | .749 |
| *Eig* | 2.37 | 2.59 | 2.69 | 2.76 | 2.02 | 2.76 | 2.09 | 2.41 | 2.45 | 2.12 | 2.77 | 2.70 |
| *% Var* | 47.5 | 51.57 | 53.8 | 55.1 | 40.4 | 55.1 | 41.8 | 48.1 | 48.9 | 42.4 | 55.3 | 54.0 |
| *Congr* | -- | .997 | .990 | .994 | .971 | .993 | .984 | .993 | .996 | .983 | .993 | .995 |
|  | ALL | **US** | **Mex** | **Bra** | **UK** | **SA** | **Ken** | **Chi** | **SK** | **Aus** | **UAE** | **Indo** |

Note: Loadings are from principal components analysis, first dimension, unrotated.

Tucker’s congruence coefficient ϕ was computed using the spreadsheet provided by DeCoster [29]. Unlike Barrett’s Orthosim program [31,32], this program does not first apply Procrustes rotation; however, there is only one factor analyzed for each country, so there is nothing to rotate. Further, congruence values for all three constructs indicate near-identical factor structure between the overall and each country-specific sample, so that Procrustes rotation is also unnecessary. Finally, we could not run the HA procedure with bootstrapping to obtain 95% CIs for the congruence values, because the sample sizes are so large that the program eventually freezes. However, all the congruence values are extremely high and the large sample sizes have tiny sample errors, so the CIs would be very narrow. Note the slight distinction between public (Q17_1 & 3) and private (Q17_2, 5 & 6) PEB loadings overall and for most countries, but a large distinction for UK and Australia.

**S3.6 Summary**

Given that, because of the secondary nature of the surveys and data,

- The specific concepts and respective items were not implemented by the researchers,
- And do not necessarily represent full or any representation of standard concepts or their measures in the literature, and
- Were not intended for the specific exploratory overall and (mis)matching tests,
- The EFA and CFA results and factor congruence tests, and
- Both Cronbach’s α and Hayes and Coutts [25] ω reliability assessment results,

these results provide good justification for the scales and subscales, across and within countries, satisfactory for our purposes.

**S4. Taking into Account Country-level Effects in the Overall Analyses**

Even though we have established consistency of the factor loadings across countries, we can still consider whether there are country-level influences that need to be taken account. Because the overall analyses include data from 11 countries, it may be necessary to consider robust standard errors (due to heterogeneity in variance in each measure across countries) and cluster robust standard errors (due to differences associated with the countries, or second-level influences/random effects in a multi-level modeling approach), or bypass those through an approach that controls for country-level influences.

Table S5 shows results from analyses of overall mean differences across countries (F-test with standard errors and Welch-test with corrected robust errors), of heterogeneity of variances across countries (Levene), and the intracluster coefficient (ICC) along with the resulting effective sample size, for the main measures. The standard error and robust error means tests are all significant at *p*<.001, and the means exhibit significant heterogeneity of variance (Levene test; all *p*<.001). However, the sample size is very large, so there will be small *p*-values even for extremely small effect sizes. The ICC values are .04 for efficacy, .06 for PEBs, and .10 for EA, reducing the overall effective sample size, though those remain very large (from 5587 to 7914). We also assessed heteroscedasticity using the Glejser test. Results were significant for pro-EA and EFF (*p*<.001). Thus, there is some country-level effect, though not large, and some reduction of effective sample size. So country-level effects may be taken into consideration. The effective sample sizes are so large that estimated power for an effect size within each country of .15 is 1.00 (using GPower).

One might consider a multi-level modeling (MLM) approach toward managing the second-level (country) influences. However, Angrist and Pischke [34] recommend at least 40 to 50 second-level units, and Bryan and Jenkins [35] show through simulations that at least 20 countries are needed for unbiased and stable errors. Further, McNeish et al. [36] argue that MLM is unnecessary in many cases, and testing for, and correcting, cluster robust standard errors is often sufficient. In addition, for our study, detailed testing would require 11 cross-level country-by-construct interactions for direct effects along for each of the three main constructs, creating 33 tests, or 11 x 8 for each of the (mis)matches, creating 88 tests.

Bryan and Jenkins [35] summarize alternatives to MLM: 1) a common model applied to pooled data using country as fixed effects, 2) a common model applied for all countries combined using cluster robust standard errors, and 3) a separate model fitted to the data for each country (used for testing the country-specific relationships). We also considered an additional common model approach 4 (Hayes Process routine) that controls for the country level effects by standardizing relevant variables within countries before conducting the overall analyses, which removes the issue of cluster standard errors, because each country has the same mean and variance due to Z-scores; while the Process routine applies the HC3 correction for heteroscedasticity (i.e., uses robust standard errors), it does not implement cluster robust standard errors.

Thus the paper reports results from approach 1) for the overall analysis (i.e., GLM with dummy country codes as a factor), and approach 3) for the country analyses (i.e., separate regressions for each country).

**Table S4.1. Tests for Overall Mean Differences, Robust Errors Mean Test, Homogeneity of Variance, and Intra-Cluster Coefficients across 11 Countries.**

| **Variable** | **Overall**  **F(10,**  **10989)** | **Adj R^2^** | **Welch**  **F(10, 4393.9, 4394.1, 4392.3)** | **Levene**  **F(10,**  **10989)** | **Glejser β** | **ICC** | **ESS** |
| --- | --- | --- | --- | --- | --- | --- | --- |
| EA | 118.24 *** | .096 | 115.79 *** | 20.78 *** | .066 *** | .097 | 5587.8 |
| EFF | 44.69 *** | .038 | 45.05 *** | 18.78 *** | -.063 *** | .039 | 7913.7 |
| PEB | 64.1 *** | .054 | 59.89 *** | 35.54 *** | -- | .055 | 7096.8 |

Note: *F*: Overall Anova means test; Welch=robust errors means test; Levene=test of homogeneity of variances; Glejser=test for heteroscedasticity of predictors; ICC=intracluster coefficient; ESS=Effective overall sample size taking into account ICC (actual overall sample N=10,999).

*** *p* < .001.

**S5. Statistical Differences across Models and Countries**

We do not conduct statistical tests of differences in coefficients across models, or across countries, for three primary reasons. First, the sample sizes are sufficiently large to enable even very small differences to achieve statistical significance. Second, all the models share subsets of the same variables, so such tests would not be independent. Third, we do not hypothesize specific differences across countries.

**S6. Definitions and Abbreviations**

**Table S6.1. Definitions and Abbreviations of Terms Used in Text.**

| **Terms** | **Categories** | **Definition** |
| --- | --- | --- |
| Models | Simple | Models (direct and moderated) tested on combined measures of EA, EFF, and PEBs |
|  | (Mis)match | Models (direct and moderated) tested on combinations of specific and general measures of EA, EFF, and PEBs |
| Domains |  | The Merriam-Webster dictionary (<https://www.merriam-webster.com/dictionary>) provides these definitions (along with others):  General: involving, applicable to, or affecting the whole; relating to, determined by, or concerned with main elements rather than limited details;  Specific: sharing or being those properties of something that allow it to be referred to a particular category; a characteristic quality or trait |
|  | Specific | Specific measures are focused on a particular object or action and typically individual and/or direct: EAC, EFFS, PEBPr |
|  | General | General measures include a broad range of objects or actions and are typically social and/or indirect: EAV, EFFC, PEBPu |
|  | Combined | Measures that involve items relevant to both specific and general domains, or, in the literature, that do not distinguish between the domains: EA, EFF, PEBs |
| Environmental attitude | EA  EAC  EAV | Environmental attitudes (combined; concern and values)  Environmental concern; typically refers to an individual’s concern about a specific environmental condition, such as air pollution  Environmental values; typically refers to a basic orientation toward nature or the environment in general |
| Pro-environmental behaviors | PEBs  PEBPr  PEBPu | Pro-environmental behaviors (combined; private and public)  Private-sphere pro-environmental behaviors  Public-sphere pro-environmental behaviors |
| Efficacy | EFF | Efficacy (combined; self- and collective) |
|  | EFFS | Self-efficacy |
|  | EFFC | Collective efficacy |
| Samples | Overall | Analysis uses the cross-country sample of N=11,000 |
|  | By Country | Analysis uses country-specific sample(s) of N=1,000 |
| Other | NGS | National Geographic Society |
|  | TPB | Theory of planned behavior |

**S7. References (for S1-S6)**

1. Goddard RD, Hoy WK, Hoy AW. Collective efficacy beliefs: Theoretical developments, empirical evidence, and future directions. Educ Res. 2004 Apr 1;33(3):3–13.

2. Landmann H, Rohmann A. Being moved by protest: Collective efficacy beliefs and injustice appraisals enhance collective action intentions for forest protection via positive and negative emotions. J Environ Psychol. 2020 Oct;71:101491.

3. Fernández-Ballesteros R, Díez-Nicolás J, Caprara GV, Barbaranelli C, Bandura A. Determinants and structural relation of personal efficacy to collective efficacy. Appl Psychol. 2002;51(1):107–25.

4. Jugert P, Greenaway KH, Barth M, Büchner R, Eisentraut S, Fritsche I. Collective efficacy increases pro-environmental intentions through increasing self-efficacy. J Environ Psychol. 2016 Dec 1;48:12–23.

5. Versland TM, Erickson JL. Leading by example: A case study of the influence of principal self-efficacy on collective efficacy. Ng DFS, editor. Cogent Educ. 2017 Jan 1;4(1):1286765.

6. Roos SM, Potgieter JC, Temane MQ. Self-efficacy, collective efficacy and the psychological well-being of groups in transition. J Psychol Afr [Internet]. 2014 May 1 [cited 2023 Jul 8]; Available from: https://www.tandfonline.com/doi/abs/10.1080/14330237.2013.10820668

7. Lent RW, Schmidt J, Schmidt L. Collective efficacy beliefs in student work teams: Relation to self-efficacy, cohesion, and performance. J Vocat Behav. 2006 Feb 1;68(1):73–84.

8. Morton TA, Rabinovich A, Marshall D, Bretschneider P. The future that may (or may not) come: How framing changes responses to uncertainty in climate change communications. Glob Environ Change. 2011 Feb;21(1):103–9.

9. Knerr S, Bowen DJ, Beresford SAA, Wang C. Genetic causal beliefs about obesity, self-efficacy for weight control, and obesity-related behaviours in a middle-aged female cohort. Psychol Health. 2016 Apr;31(4):420–35.

10. Berger IE, Corbin RM. Perceived consumer effectiveness and faith in others as moderators of environmentally responsible behaviors. J Public Policy Mark. 1992 Sep;11(2):79–89.

11. Kim Y. Understanding green purchase: The influence of collectivism, personal values and environmental attitudes, and the moderating effect of perceived consumer effectiveness. Seoul J Bus. 2011 Jun;17(1):65–92.

12. Lee JA, Holden SJS. Understanding the determinants of environmentally conscious behavior. Psychol Mark. 1999;16(5):373–92.

13. Meinhold JL, Malkus AJ. Adolescent environmental behaviors: Can knowledge, attitudes, and self-efficacy make a difference? Environ Behav. 2005 Jul 1;37(4):511–32.

14. Oh J, Sudarshan S, Jin E, Nah S, Yu N. How 360-degree video influences content perceptions and environmental behavior: The moderating effect of environmental self-efficacy. Sci Commun. 2020 Aug 1;42(4):423–53.

15. Walton T, Austin DM. Pro-environmental behavior in an urban social structural context. Sociol Spectr. 2011 Apr 22;31(3):260–87.

16. Anker AE, Feeley TH, Kim H. Examining the attitude–behavior relationship in prosocial donation domains. J Appl Soc Psychol. 2010;40(6):1293–324.

17. Hurst Loo AM, Walker BR. Climate change knowledge influences attitude to mitigation via efficacy beliefs. Risk Anal [Internet]. [cited 2022 Sep 26];n/a(n/a). Available from: https://onlinelibrary.wiley.com/doi/abs/10.1111/risa.14026

18. Green DP, Ha SE, Bullock JG. Enough already about “black box” experiments: Studying mediation is more difficult than most scholars suppose. Ann Am Acad Pol Soc Sci. 2010 Mar 1;628(1):200–8.

19. Fiedler K, Harris C, Schott M. Unwarranted inferences from statistical mediation tests – An analysis of articles published in 2015. J Exp Soc Psychol. 2018 Mar;75:95–102.

20. Hu L, Bentler PM. Cutoff criteria for fit indexes in covariance structure analysis: Conventional criteria versus new alternatives. Struct Equ Model Multidiscip J. 1999 Jan 1;6(1):1–55.

21. Kline RB. Principles and practice of structural equation modeling. 3rd ed. Guilford Press; 2010.

22. Gaskin J, Lim J. Model fit measures [Internet]. AMOS Plugin. 2016 [cited 2023 Jul 8]. Available from: http://statwiki.gaskination.com/index.php?title=Main_Page

23. Fornell C, Larcker DF. Evaluating structural equation models with unobservable variables and measurement error. Journal of Marketing Research. 1981;18(1):39–50.

24. ADANCO 2.3 [Internet]. Kleve, Germany; 2021 [cited 2023 Nov 22]. (Composite Modeling). Available from: http://www.composite-modeling.com/

25. Hayes AF, Coutts JJ. Use omega rather than cronbach’s alpha for estimating reliability. But…. Commun Methods Meas. 2020 Jan 2;14(1):1–24.

26. Cho E. Neither cronbach’s alpha nor mcdonald’s omega: A commentary on sijtsma and pfadt. Psychometrika. 2021 Dec 1;86(4):877–86.

27. Lorenzo-Seva U, ten Berge JMF. Tucker’s congruence coefficient as a meaningful index of factor similarity. Methodol Eur J Res Methods Behav Soc Sci. 2006;2(2):57–64.

28. Milfont TL, Bain PG, Kashima Y, Corral-Verdugo V, Pasquali C, Johansson LO, et al. On the relation between social dominance orientation and environmentalism: A 25-nation study. Soc Psychol Personal Sci. 2018 Sep 1;9(7):802–14.

29. DeCoster J. Congruence coefficients for factor analysis 2010-02-08 [Internet]. 2010 [cited 2023 Jul 8]. Available from: http://www.stat-help.com/

30. Błachnio A, Przepiorka A, Benvenuti M, Cannata D, Ciobanu AM, Senol-Durak E, et al. Cultural and personality predictors of facebook intrusion: A cross-cultural study. Front Psychol [Internet]. 2016 [cited 2023 Jul 8];7. Available from: https://www.frontiersin.org/articles/10.3389/fpsyg.2016.01895

31. Barrett P. Orthosim v3 [Internet]. 2022 [cited 2022 Jun 18]. Available from: https://www.pbarrett.net/orthosim/orthosim.html

32. Barrett P. Orthosim target-comparison matrix fitting [Internet]. 2013. Available from: https://imaging.mrc-cbu.cam.ac.uk/statswiki/FAQ/congruenceC?action=AttachFile&do=get&target=congruence.pdf

33. Lovik A, Nassiri V, Verbeke G, Molenberghs G. A modified tucker’s congruence coefficient for factor matching. Methodology. 2020 Apr 6;16(1):59–74.

34. Angrist JD, Pischke JS. Mostly harmless econometrics [Internet]. 2009 [cited 2023 Jul 8]. 392 p. Available from: https://press.princeton.edu/books/paperback/9780691120355/mostly-harmless-econometrics

35. Bryan ML, Jenkins SP. Multilevel modelling of country effects: A cautionary tale. Eur Sociol Rev. 2016 Feb 1;32(1):3–22.

36. McNeish D, Stapleton LM, Silverman RD. On the unnecessary ubiquity of hierarchical linear modeling. Psychol Methods. 2017;22(1):114–40.
